# Mathematical modeling of HIV-1 transmission risk from condomless anal intercourse in HIV-infected MSM by the type of initial ART

**DOI:** 10.1371/journal.pone.0219802

**Published:** 2019-07-19

**Authors:** Juan Berenguer, Javier Parrondo, Raphael J. Landovitz

**Affiliations:** 1 Hospital General Universitario Gregorio Marañón, Madrid, Spain; 2 Instituto de Investigación Sanitaria Gregorio Marañón (IiSGM), Madrid, Spain; 3 Parrondo Health, Coslada, Spain; 4 UCLA Center for Clinical AIDS Research & Education, Los Angeles, CA, United States of America; Fundación Huésped ARGENTINA

## Abstract

**Background:**

Initiation of antiretroviral therapy (ART) for HIV infection using regimens that include integrase strand transfer inhibitors (INSTIs) is associated with a faster decline in HIV-1 RNA than what is observed with regimens that are anchored by other ART drug classes. We compared the impact of ART regimens that include dolutegravir (DTG), raltegravir (RAL), efavirenz (EFV), or darunavir/ritonavir (DRV/r), in treatment naïve men who have sex with men (MSM) on the probability of HIV-1 sexual transmission events (HIV-TE).

**Setting:**

Mathematical model.

**Methods:**

We used discrete event simulation modeling to estimate HIV-TE during the first 8 weeks after initiation of ART. HIV-1 RNA decay in men was modeled from the databases of three clinical trials: Single (DTG vs. EFV), Spring-2 (DTG vs. RAL) and Flamingo (DTG vs. DRV/r).

**Results:**

All regimens substantially reduced the number of HIV-TE compared to no treatment. DTG led to fewer HIV-TE than its comparator in each of the three trials: 22.72% fewer transmissions than EFV; 0.52% fewer transmissions than RAL; and 38.67% fewer transmissions than DRV/r. The number of patients needed to treat with DTG to prevent one transmission event instead of comparator was 48 vs EFV, 2,194 vs RAL, and 31 vs DRV/r.

**Conclusion:**

Unsurprisingly, this mathematical model showed that all regimens reduced HIV-TE compared to no treatment. The results also suggest that that initial use of INSTIs, by virtue of their superior viral decay kinetics, have the potential to reduce HIV-1 horizontal transmission following initiation of ART in naïve MSM.

**Trial registration:**

ClinicalTrials.gov NCT03183154.

## Introduction

Men who have sex with men (MSM) are at disproportionate risk of HIV-1 infection, as are people who inject drugs, transgender individuals, and commercial sex workers [1]. The HIV-1 epidemic among MSM is characterized by persistently increased incidence, even as incidence in other populations has declined [2]. Epidemic persistence among MSM is at least partially explained by the high per-act and per-partner probability of HIV-1 transmission associated with receptive anal sex [3, 4].

To control or eliminate HIV-1 spread among MSM, robust prevention strategies are required. In addition to condom use, these include the early initiation of antiretroviral therapy (ART) among HIV-infected individuals to reduce the rates of transmission of HIV-1 to sexual partners [5–7], and the implementation of pre-exposure prophylaxis (PrEP) to prevent the acquisition of HIV-1 infection in uninfected individuals [8–10].

Initiation of ART with regimens that include integrase strand transfer inhibitors (INSTIs) is associated with a more rapid decline in HIV-1 RNA than with regimens that include non-nucleoside reverse transcriptase inhibitors (nnRTIs) or protease inhibitors (PIs). The clinical implications of this observation are unknown, although recent clinical trials showed that in women starting ART late in gestation, INSTI-based ART achieved more rapid virological suppression before delivery compared to efavirenz (EFV), although no significant differences in the number of vertical transmission of HIV were found between treatment arms [11, 12].

A clinical trial to evaluate the differential impact of first ART initiation with various regimens in MSM on the probability of sexual transmission of HIV-1 would be infeasible; hence we aimed to evaluate this issue using a mathematical model.

## Materials and methods

**Fig 1:**
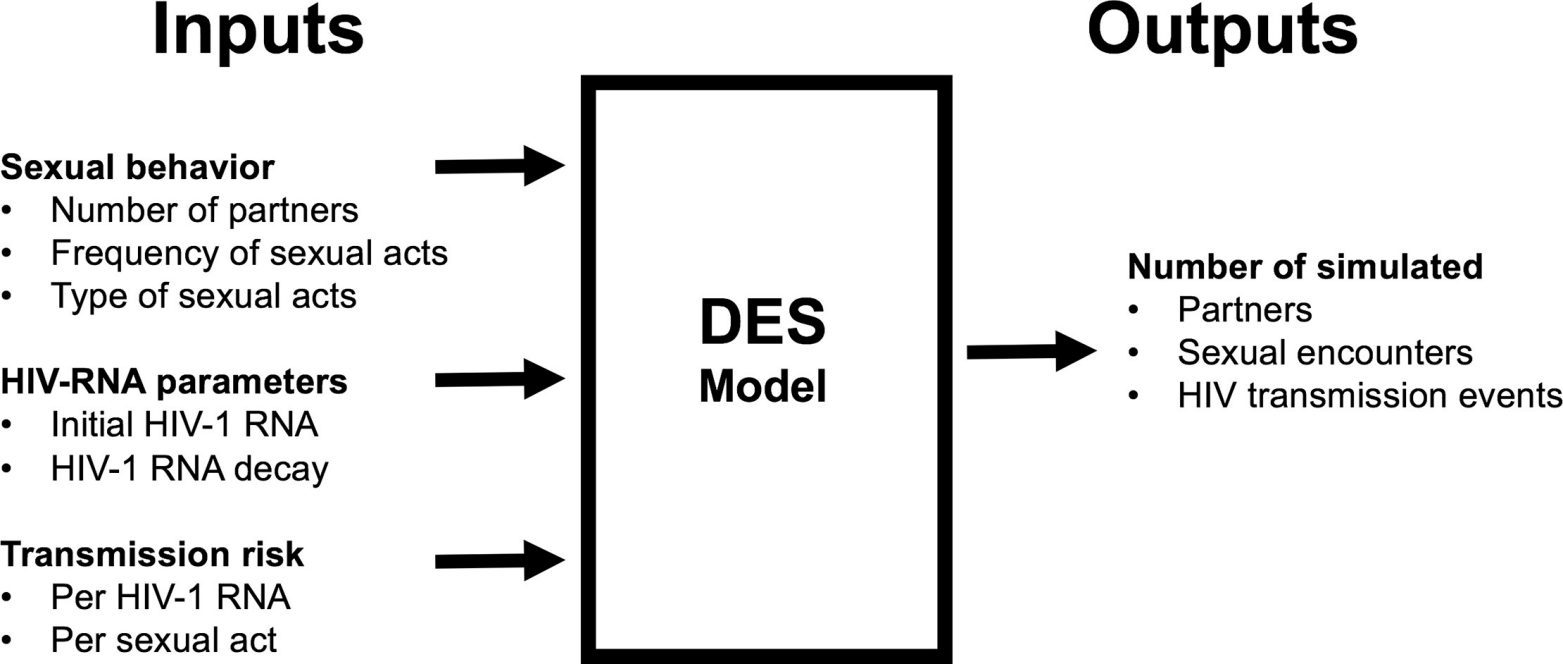
Key input and output variables in the model. Abbreviations: DES, discrete simulation events.

We modeled the probability of sexually transmitted HIV-1 infection during the first 8 weeks after initiation of ART in naïve HIV-infected MSM with chronic HIV-infection. The probability of HIV-1 sexual transmission depends on per-coital act rates of HIV-1 transmission, sex behavior types and frequency, and viral burden (Fig 1).

### Structure of the model

A probabilistic approach was used to develop a discrete event simulation (DES) model using Microsoft Excel (2016) and Visual Basic for applications (VBA) to determine the number of anticipated sexual transmission events at each timepoint at and after ART initiation for each treatment scenario (Fig A in S1 File [Technical Appendix]).

Five million theoretical individuals were modeled to determine the number of secondary sexually transmitted HIV-1 infections arising from MSM initiating dolutegravir [DTG]-containing ART regimens versus infections arising from individuals starting ART containing comparator regimens (efavirenz [EFV], raltegravir [RAL], or darunavir/ritonavir [DRVr]-based ART) and versus no treatment.

The data for virologic decay was modeled based on the individual patient level data from the pivotal phase III trials of the INSTI dolutegravir (DTG): Single [13], Spring-2 [14], and Flamingo [15]. We determined the number of sexually transmitted HIV-1 infections from theoretical patients receiving ART containing DTG versus each trial comparator: the nnRTI efavirenz (EFV) in Single, the INSTI raltegravir (RAL) in Spring-2, and the PI darunavir/ritonavir (DRV/r) in Flamingo, and versus no treatment. Each theoretical patient was cloned to obtain nine identical patients who were exposed to no therapy (in all three studies), and to ART containing DTG (all three studies), EFV (Single), RAL (Spring-2) and DRV/r (Flamingo). At the beginning of the simulation, time and transmission event counter variables were set to 0, and four attributes were randomly set for each simulated patient: the HIV-1 RNA decay curve for each simulated patient’s ART treatment regimen, the number of sexual partners during the period of interest, the number of sexual encounters per partner, and the type and the timing of each sexual encounter. HIV-1 RNA for each clone was modelled to decay following a fractional polynomial regression (see below) obtained randomly from the observed decay kinetics of the relevant clinical trial and treatment regimen. For the untreated clones, we assumed that the baseline HIV-1 RNA in untreated patients remained stable during the entire observation period. Each time that a sexual encounter occurred, the probability of HIV-1 transmission to the sexual partner was modeled based on the type of sexual exposure and the HIV-1 RNA at the time of the encounter. If the partner became infected as a result of the sexual encounter, this was recorded in a counter variable, and the simulation proceeded to assess a new partner. The process was repeated for each partner until the end of the simulation time horizon when the time variable was reset to 0 to continue the simulation with the next cloned patient.

### Model inputs

#### Sexual behavior parameters

The sexual behavior during the first 8 weeks after initiation of ART, including the number of HIV-negative sexual partners, and the frequency of condomless insertive and receptive anal intercourse per partner was simulated from the MSM population in the INSIGHT Strategic Timing of AntiRetroviral Treatment (START) Trial [16]. All participants in the START trial completed a risk behaviour questionnaire that assessed condomless sex with serodifferent partners in the two months prior to enrolment in the trial. In the START population, 20% of MSM reported condomless sex with an HIV-discordant status partner in the 8 weeks prior to randomization (See full description of sexual behaviour in Table A and Table B in S1 File).

#### Viral load parameters

Fractional polynomial regression of repeated measurements of HIV-1 RNA from baseline up to week 24 from the databases of Single, Spring-2, and Flamingo were used to model HIV-1 RNA decay curves for each simulated patient and ART regimen [17, 18], (S1 File). HIV-1 RNA measurements were log_10_ transformed to stabilize the variance and to meet normality assumptions of the residuals.

The initial HIV-1 RNA of each simulated patient was obtained from the randomly generated distribution (fractional polynomial regression) of the HIV-1 RNAs from patients included in the clinical trials. (S1 File). The patients were categorized according to baseline viral load (<10,000 copies/ml, 10,000 to <100,000 copies/ml, and > 100,000 copies/ml).

#### Transmission risk per-sexual exposure act

The HIV-1 transmission rates per sexual exposure by HIV-1 RNA were obtained from the Wilson mathematical model [19], in which the risk of transmission of HIV-1 based on HIV-1 RNA was modeled from the results of the Rakai study of HIV transmission in heterosexual couples [20]. On the basis of the Rakai data, each ten-fold increment in viral load is associated with a 2.45-fold (95% CI 1.85–3.26) increase in the risk of HIV transmission per sexual contact, as expressed by the equation:

$$\beta_{1}=2.45^{\log_{10}}\left(V_{1}/V_{0}\right)\beta_{0}$$

where β_0_ is the probability of HIV transmission from a person with a baseline viral load V_0_, and β_1_ is the transmission probability corresponding to any other viral load V_1_, whether above or below the baseline. V_0_ (lower and upper uncertainty bounds) is 4.3 x 10^−5^ (1.6 x 10^−5^–11.6 x 10^−5^) and corresponds to the expected transmission probability per male to female sexual act in a serodiscordant partnership, assuming the HIV-infected male has a viral load of 10 copies per ml. As the Wilson equation used was done to estimate the risk of HIV transmission at a given HIV-1 RNA among serodiscordant heterosexual couples, the probability was modified by using the Odds Ratio of the type of sexual relationship (receptive or insertive anal intercourse) versus a receptive vaginal intercourse that were obtained from a recent systematic review by Patel et al. [21] (S1 File).

We assumed that sexual behavior did not change during the 8 weeks subsequent to the initiation of ART and did not consider the use of PrEP or the presence/impact of untreated sexually transmitted infections.

### Model outcomes

For each treatment arm in Single, Spring-2, and Flamingo, the model outputs provide the following parameters for the simulated patients engaging in condomless sex with an HIV-discordant status partner (approximately 20% of the total population) : 1) The number of simulated HIV-1-negative partners, 2) The number of simulated sexual encounters, and 3) The number of simulated HIV-1 transmission events. From these outcomes, the proportion of simulated HIV-1 transmission events for each treatment arm compared with no therapy was calculated. We also calculated the number of patients needed to treat (NNT) for DTG compared to EFV, RAL, or DRV/r-based ART in order to prevent one HIV-1 infection [22].

### Sensitivity analyses

Given the uncertainty of the β_0_ parameter in Wilson equation, two sensitivity analyses were performed with the lower and upper 95% confidence interval (CI) values of this parameter. In addition, we also conducted six additional sensitivity analyses considering the transmission effects over the horizon of week 0 to week 24, with two different asumptions for the sexual activity during this extended period based on the 8 week data reported in the START trial, and with the three different probabilities of infection according to the β_0_ parameter of the Wilson equation (mean value and lower and upper 95% CI values) (S1 File).

## Results

### Simulated sexual activity

The simulated sexual activity over the full 0 to 8-week period after initiation of ART in the three arms corresponding to the Single, Spring-2, and Flamingo trials is shown in Table 1.

**Table 1:** Simulated sexual activity and HIV-1 transmission events after initiation of ART, for the full week 0 to 8 period, in the three treatment arms corresponding to the Single, Spring-2, and Flamingo trials parametrized according to the sexual risk behavior questionnaire in MSM recruited in the START trial.

	Base case scenario ^a^			Sensitivity analysis 1 ^a^			Sensitivity analysis 2 ^a^		
Simulated sexual activity ^b^	Single	Spring-2	Flamingo	Single	Spring-2	Flamingo	Single	Spring-2	Flamingo
Patients who initiated ART	5,000,000	5,000,000	5,000,000	5,000,000	5,000,000	5,000,000	5,000,000	5,000,000	5,000,000
Patients who engaged in CLS-D (20% of those initiating ART)	1,000,000	1,000,000	1,000,000	1,000,000	1,000,000	1,000,000	1,000,000	1,000,000	1,000,000
Partners of patients who engaged in CLS-D	1,787,964	1,810,363	1,826,137	1,782,200	1,802,297	1,832,544	1,784,271	1,802,013	1,834,088
Sexual encounters in patients who engaged in CLS-D	7,812,258	7,876,108	7,641,026	7,808,892	7,849,296	7,637,826	7,816,255	7,867,745	7,642,580
Partners per patient who engaged in CLS-D	1.79	1.81	1.83	1.78	1.80	1.83	1.78	1.80	1.83
Sexual encounters per partner in patients who engaged in CLS-D	4.37	4.35	4.18	4.38	4.36	4.17	4.38	4.37	4.17
**Simulated HIV-1 transmission events**	**No cART**	**No cART**	**No cART**	**No cART**	**No cART**	**No cART**	**No cART**	**No cART**	**No cART**
New infections	809,773	771,996	733,166	655,940	617,982	561,346	892,542	871,906	855,448
HIV-1 transmission events per 100 patients who initiated ART	16.20	15.44	14.66	13.12	12.36	11.23	17.85	17.44	17.11
HIV-1 transmission events per 100 patients who engaged in CLS-D	80.98	77.20	73.32	65.59	61.80	56.13	89.25	87.19	85.54
HIV-1 transmission events per 100 partners (CLS-D)	45.29	42.64	40.15	36.81	34.29	30.63	50.02	48.39	46.64
	**DTG**	**DTG**	**DTG**	**DTG**	**DTG**	**DTG**	**DTG**	**DTG**	**DTG**
New infections	11,823	6,905	7,135	4,144	2,350	2,751	32,965	18,453	18,976
HIV-1 transmission events per 100 patients who initiated ART	0.24	0.14	0.14	0.08	0.05	0.06	0.66	0.37	0.38
HIV-1 transmission events per 100 patients who engaged in CLS-D	1.18	0.69	0.71	0.41	0.24	0.28	3.30	1.85	1.90
HIV-1 transmission events per 100 partners (CLS-D)	0.66	0.38	0.39	0.23	0.14	0.15	1.85	1.02	1.03
Proportion of HIV-1 transmission events compared with no Rx	0.01	0.01	0.01	0.01	<0.01	<0.01	0.04	0.02	0.02
	**EFV**	**RAL**	**DRV/r**	**EFV**	**RAL**	**DRV/r**	**EFV**	**RAL**	**DRV/r**
New infections	195,797	10,910	290,614	97,073	4,006	174,954	311,538	27,372	424,170
HIV-1 transmission events per 100 patients who initiated ART	3.92	0.22	5.81	1.94	0.08	3.50	6.23	0.55	8.48
HIV-1 transmission events per 100 patients who engaged in CLS-D	19.58	1.09	29.06	9.71	0.40	17.50	31.15	2.74	42.44
HIV-1 transmission events per 100 partners (CLS-D)	10.95	0.60	15.91	5.45	0.22	9.55	17.46	1.52	23,13
Proportion of HIV-1 transmission events compared with no Rx	0.24	0.01	0.40	0.15	0.01	0.31	0.35	0.03	0.50
**NNT with DTG instead of comparator to prevent one infection**	**48**	**2194**	**31**	**95**	**5,952**	**52**	**32**	**984**	**22**

^a^Base case scenario: probability of transmission according to the mean value of the β_0_ parameter in the Wilson equation. Sensitivity analysis 1: probability of transmission according to the lower 95% confidence interval value of the β_0_ parameter in the Wilson equation. Sensitivity analysis 2: probability of transmission according to the upper 95% confidence interval value of the β_0_ parameter in the Wilson equation.

^b^ MSM population is based in sexual activity report on START trial (only 20% of the MSM population have condomless sex with an HIV-1-discordant status partner). In addition, a very small number of intercourse events among MSM in the START trial were reported to be with women

Abbreviations: cART, combination antiretroviral therapy; MSM, men who have sex with men; CLS-D, condomless sex with an HIV-1-discordant status partner; MSM Male who have sex with males; DTG, dolutegravir; EFV, efavirenz; DRV/r, darunavir/ritonavir; NNT, number needed to treat.

Overall, during the 8 weeks after the initiation of ART in the three clinical trials, per 5 million simulated MSM patients initiating ART, 1 million (20%) engaged in condomless sex; those patients had approximately 1.8 million total simulated sexual partners, resulting in 7.6–7.9 million simulated condomless sex acts. The number of simulated condomless sex acts with an HIV-negative partner per simulated patient who engaged in condomless sex was approximately 4.2–4.4 in the three trials.

### Simulated HIV-1 transmission events

The number of new simulated HIV-1 transmission events during the full 0 to 8-week period after initiation of ART in the three arms corresponding to the Single, Spring-2, and Flamingo trial is shown in Table 1. Overall, during the 8 weeks after the initiation of ART using data from the Single trial, the number of new simulated transmitted HIV-1 infections per 100 patients was 16.2 with no treatment, 0.24 in the DTG arm, and 3.92 in the EFV arm. Using data from the Spring-2 trial, the number of new simulated transmitted infections per 100 patients was 15.44 with no treatment, and 0.14 in the DTG arm, and 0.22 in the RAL arm. Using data from the Flamingo trial, the number of new simulated transmitted infections per 100 patient was 14.66 with no treatment, 0.14 in the DTG arm, and 5.81 in the DRV/r arm.

### Reduction of HIV-1 transmission events

The proportion of new simulated HIV transmission events in the full 0 to 8-week period after initiation of cART, and in the three arms of the three clinical trials compared with no treatment is shown in Table 1. Overall, during the 8 weeks after the initiation of ART in the Single trial, the relative number of new simulated HIV-1 infections compared to no treatment was 0.01 with DTG and 0.24 with EFV. In the Spring-2 trial, the relative number of new simulated transmitted infections compared to no treatment was 0.01 with both DTG, and RAL. In the Flamingo trial, the relative number of new simulated transmitted infections compared to no treatment was 0.01 with DTG, and 0.40 with DRV/r (Table 1).

**Fig 2:**
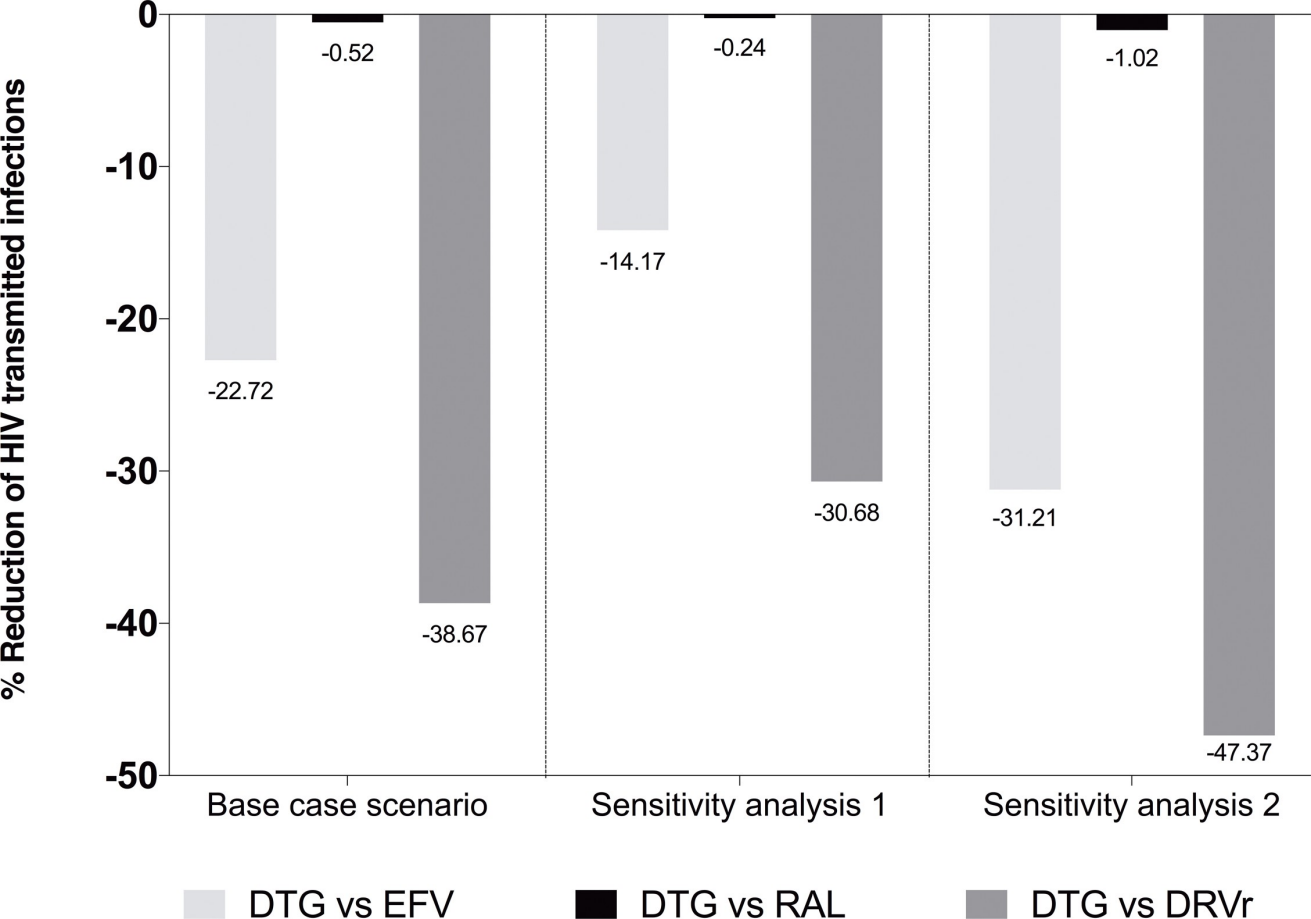
Comparison between DTG and comparators (EFV in Single, RAL in Spring-2, and DRV/r in Flamingo) in the relative reduction of new simulated sexually transmitted infections in comparison to no treatment for the full 0 to week 8 period. Base case scenario: probability of transmission according to the mean value of the β_0_ parameter in the Wilson equation. Sensitivity analysis 1: probability of transmission according to the lower 95% confidence interval value of the β_0_ parameter in the Wilson equation. Sensitivity analysis 2: probability of transmission according to the upper 95% confidence interval value of the β_0_ parameter in the Wilson equation. Abbreviations: DTG, dolutegravir; RAL, raltegravir; DRVr, darunavir/ritonavir.

Although all ART regimens in the three clinical trials substantially reduced the number of new simulated sexually transmitted infections compared to no treatment, during the 8 weeks after initiation of ART, DTG led to fewer new simulated transmitted infections than its comparator in each of the three trials: 22.72% fewer transmissions than EFV in Single, 0.52% fewer transmissions than RAL in Spring-2, and 38.67% fewer transmissions than DRV/r in Flamingo (Fig 2).

The number of patients needed to be treated with DTG instead of EFV, RAL, and DRV/r in order to prevent one single infection in the Single, Spring-2, and Flamingo trials were 48, 2,194, and 31, respectively (Table 1).

### Sensitivity analyses

The simulated sexual activity, HIV-1 transmission events, and NNT with DTG instead of comparator to prevent one infection, in the in the three treatment arms corresponding to the Single, Spring-2, and Flamingo trials taking into account the lower and upper uncertainty bounds for expected transmission probabilities according to the 95% CI values of the β_0_ parameter in the Wilson equation are shown in Table 1. The results of the six additional sensitivity analyses considering the transmission effects from week 0 to week 24 are shown in the Technical Appendix (S1 File).

## Discussion

Virologic suppression from combination ART renders HIV-infected individuals non-infectious, and has revolutionized both the HIV-treatment and HIV-prevention paradigms. The HPTN 052, PARTNER, PARTNER-2, and Opposites Attract studies have provided the data that virologic suppression essentially eliminates sexual transmission.[5, 6, 23–25] However, virologic suppression is not an immediate result of ART initiation, leaving a window of vulnerability for ongoing sexual transmission in the peri-ART initiation period.

In this modelling analysis, we demonstrate the translation of this observation to a transmission model that attempts to estimate the clinical and public health sequelae of this period of vulnerability. We used discrete event simulation modeling to estimate sexually transmitted HIV-1 infections during the first 8 weeks after initiation of ART with four different regimens based on DTG, EFV, RAL, and DRV/r. We selected a time horizon of 8 weeks in our primary analysis because this is the period from which we have data about self-reported sexual activity among HIV-1 infected individuals coming from clinical trials [16], and also because marked differences in viral load decay between INSTI-based therapy and non-INSTI-based therapies occur in this period. We modelled 5 million theoretical patients to guarantee that 1 million (20%) engaged in condomless condomless sex acts with an HIV-negative partner in order to ensure model stability [26].

The observation that HIV-1 viral decay is more rapid after first initiation of INSTI-based rather than with nnRTI-based and PI-based ART led us to hypothesize that there would be measurable differences in sexual transmission attributable to the use of DTG-based ART and EFV, DRV/r, or even RAL-based ART. Given that the differential viral decay rates of each initial treatment regimen, it would be intuitive that these rates would translate into differential numbers of secondary sexual transmission events; to the best of our knowledge, this modelling exercise is the first attempt to estimate the magnitude and limitations of such differences. In an era of rapidly evolving changes to first-line treatment guidelines globally, particularly if differential impact could be shown for nnRTI vs. INSTI-based initial ART, findings could help motivate expeditious switching of first-line recommendations to INSTI-based therapy. While it has been hypothesized that protease inhibitors in particular may preferentially render residual viral particles in plasma and/or genital secretions non-replicaiton competent, the contribution of this phenomenon to non-transmissability has not been established. Indeed, HPTN 052, Opposites Attract, PARTNER, and PARTNER-2 were not powered to distinguish effects by regimen, highlighting the importance of modelled data to dissect early differences between commonly used first-line regimens.

Our most important finding is a confirmatory one: All regimens substantially reduced the number of new sexually transmitted HIV-1 infections from HIV-infected MSM initiating ART compared to the expected number of transmissions absent ART treatment. However, we did find differences between regimens that are of interest. For 100 simulated MSM if left untreated, approximately 15 new infections were transmitted over 8 weeks in each of the three trial-based model scenarios. Over the first 8 weeks of ART containing DTG or RAL for these simulated MSM, the number of simulated HIV-1 transmission events was reduced by 99.90%, whereas EFV and DRV/r reduced the number of simulated transmission events by 76.00% and 60.00%, respectively. Between arm differences between DTG and EFV, RAL, and DRV/r were 22.72%, 0.52%, and 38.67%, respectively, all with fewer infections in the DTG-treated simulated patients than the comparators.

These differences in simulated HIV-1 transmission events between arms highlight a public health call-to-action for rapid if not immediate ART initiation upon diagnosis–particularly for sexually active MSM populations–with ART capable of reducing plasma virus loads as rapidly as possible. Choice of first line ART agents as part of national guidelines could have a significant impact on population-level incidence in regions where an MSM epidemic predominates.

These data have limitations. In our model, only HIV negative partners were modeled from the data from the START trial, without taking into account the HIV prevalence in the population. We did not consider PrEP use among sero-negative partners, which if used widely and appropriately could attenuate HIV transmissions overall, likely overwhelming the relative benefit of differences afforded by early suppression for the HIV-infected individual. Appropriately deployed PrEP services also increase rates of diagnosis and treatment of bacterial STI’s, further decreasing susceptibility of HIV-infected partners–and also not accounted for in our model. Our model assumed no changes in numbers of partners or rates of condomless sex. If individuals experiencing more rapid declines in viral load were to be aware of these laboratory results and commensurately increase their numbers of partners or decrease condom use, transmission events could be substantially increased, attenuating or abrogating the protective effects of early virus load decline. Conversely, patients being appropriately counseled as to the increased risk of HIV transmission from HIV-infected individuals before or early in the course of their ART treatment may decrease partners and/or increase condom use in the setting of diagnosis and treatment initiation. Such protective actions would be expected to mitigate the effect difference seen between regimens. Number of new infections transmitted from individuals who are aware of their diagnosis and newly initiating ART likely varies widely by region, and in some settings may represent a relatively minor proportion of new infections, particularly in comparison to infections deriving from individuals who are acutely infected and/or unaware of their diagnoses. These geographic or regional differneces may be compounded by different rates of overall viral suppression at the population level, PrEP use, and numbers of partners. The model has significant strengths as well, including inputs from real-world clinical trial data and using it to model populations of sexually active MSM whose sexual behaviors are also modeled on data from a large and geographically diverse randomized clinical trial of first-time ART initiation. In addition, the sensitivity analyses results show the robustness of the model with regard to a wide range of values in the model inputs and assumptions.

## Conclusions

In addition to context-specific efficacy and safety, cost, acceptability, and supply chain issues contribute to regional and country-specific guidelines for first-line ART treatment of persons living with HIV. Our data provide additional rationale for the evolution of first-line ART globally to include INSTI-based regimens: the potential to decrease horizontal transmissions immediately subsequent to ART initiation due to rapid decreases in viremia.

## Supporting information

S1 File Table A. Condomless sex with an HIV-1-discordant status partner in men who have sex with men (MSM). Table B. Numbers of partners according to type of condomless sex with an HIV-1-discordant status partner (CLS-D) in 459 MSM participants reporting at least one episode of CLS-D. Table C. Viral load on screening (group <10,000 copies/mL). Table D. Viral load on screening (group ≥10,000 to < 100,000 copies/mL). Table E. Viral load on screening (group ≥100,000 copies/mL). Table F. Estimated per-act probability of acquiring HIV-1 from an infected source, by sexual act. Table G. Sensitivity analyses 3–5. Simulated sexual activity and HIV-1 transmission events after initiation of ART, for the full week 0 to 24 period, in the three treatment arms corresponding to the Single, Spring-2, and Flamingo trials parametrized according to the sexual risk behavior questionnaire in MSM recruited in the START trial. Table H. Sensitivity analyses 6–8. Simulated sexual activity and HIV-1 transmission events after initiation of ART, for the full week 0 to 24 period, in the three treatment arms corresponding to the Single, Spring-2, and Flamingo trials parametrized according to the sexual risk behavior questionnaire in MSM recruited in the START trial. Figure A. Simulation Flow. Figure B. STATA results for SINGLE <10,000 copies/mL. Figure C. EFV treated patient. Simulated Log10 HIV-RNA over 24 weeks SINGLE <10,000 copies/mL (MEAN ± 95%CI). Figure D. DTG treated patient. Simulated Log10 HIV-RNA over 24 weeks SINGLE <10,000 copies/mL (MEAN ± 95%CI). Figure E. STATA results for SINGLE ≥ 10,000 to <100,000 copies/mL. Figure F. EFV treated patient. Simulated Log10 HIV-RNA over 24 weeks SINGLE ≥ 10,000 to <100,000 copies/mL (MEAN ± 95%CI). Figure G. DTG treated patient. Simulated Log10 HIV-RNA over 24 weeks SINGLE ≥ 10,000 to <100,000 copies/mL (MEAN ± 95%CI). Figure H. STATA results for SINGLE ≥ 100,000 copies/mL. Figure I. EFV treated patient. Simulated Log10 HIV-RNA over 24-weeks SINGLE ≥ 100,000 copies/mL (MEAN ± 95%CI). Figure J. DTG treated patient. Simulated Log10 HIV-RNA over 24-weeks SINGLE ≥ 100,000 copies/mL (MEAN ± 95%CI). Figure K. STATA results for SPRING2 <10,000 copies/mL. Figure L. RAL treated patient. Simulated Log10 HIV-RNA over 24-weeks SPRING2 <10,000 copies/mL (MEAN ± 95%CI). Figure M. DTG treated patient. Simulated Log10 HIV-RNA over 24-weeks SPRING2 <10,000 copies/mL (MEAN ± 95%CI). Figure N. STATA results for SPRING2 ≥ 10,000 to <100,000 copies/mL. Figure O. RAL treated patient. Simulated Log10 HIV-RNA over 24 weeks SPRING2 ≥ 10,000 to <100,000 copies/mL (MEAN ± 95%CI). Figure P. DTG treated patient. Simulated Log10 HIV-RNA over 24 weeks SPRING2 ≥ 10,000 to <100,000 copies/mL (MEAN ± 95%CI). Figure Q. STATA results for SPRING2 ≥ 100,000 copies/mL. Figure R. RAL treated patient. Simulated Log10 HIV-RNA over 24-weeks SPRING2 ≥ 100,000 copies/mL (MEAN ± 95%CI). Figure S. DTG treated patient. Simulated Log10 HIV-RNA over 24-weeks SPRING2 ≥ 100,000 copies/mL (MEAN ± 95%CI). Figure T. STATA results for FLAMINGO < 10,000 copies/mL. Figure U. DRVr treated patient. Simulated Log10 HIV-RNA over 24-weeks FLAMINGO <10,000 copies/mL (MEAN ± 95%CI). Figure V. DTG treated patient. Simulated Log10 HIV-RNA over 24-weeks FLAMINGO <10,000 copies/mL (MEAN ± 95%CI). Figure W. STATA results for FLAMINGO ≥ 10,000 to <100,000 copies/mL. Figure X. DRVr treated patient. Simulated Log10 HIV-RNA over 24-weeks FLAMINGO ≥ 10,000 to <100,000 copies/mL (MEAN ± 95%CI). Figure Y. DTG treated patient. Simulated Log10 HIV-RNA over 24-weeks FLAMINGO ≥ 10,000 to <100,000 copies/mL (MEAN ± 95%CI). Figure Z. STATA results for FLAMINGO ≥ 100,000 copies/mL. Figure AA. DRVr treated patient. Simulated Log10 HIV-RNA over 24-weeks DRVr FLAMINGO ≥ 100,000 copies/mL (MEAN ± 95%CI). Figure AB. DTG treated patient. Simulated Log10 HIV-RNA over 24-weeks DTG FLAMINGO ≥ 100,000 copies/mL (MEAN ± 95%CI). Figure AC. DTG treated patient. Simulated Log10 HIV-RNA over 24-weeks. Comparison of DTG MEAN in the three studies <10,000 copies/mL. Figure AD. DTG treated patient. Simulated Log10 HIV-RNA over 24-weeks. Comparison of DTG MEAN in the three studies ≥ 10,000 to <100,000 copies/mL. Figure AE. DTG treated patient. Simulated Log10 HIV-RNA over 24-weeks. Comparison of DTG MEAN in the three studies ≥ 100,000 copies/mL.
(DOCX)
