# Supplementary material for: Mathematical modeling of HIV-1 transmission risk from condomless anal intercourse in HIV-infected MSM by the type of initial ART
Source: PLoS One. 2019 Jul 19;14(7):e0219802. doi: 10.1371/journal.pone.0219802 (PMC6641501; doi:10.1371/journal.pone.0219802)
Supplement: S1 File — Table A. Condomless sex with an HIV-1-discordant status partner in men who have sex with men (MSM). Table B. Numbers of partners according to type of condomless sex with an HIV-1-discordant status partner (CLS-D) in 459 MSM participants reporting at least one episode of CLS-D. Table C. Viral load on screening (group <10,000 copies/mL). Table D. Viral load on screening (group ≥10,000 to < 100,000 copies/mL). Table E. Viral load on screening (group ≥100,000 copies/mL). Table F. Estimated per-act probability of acquiring HIV-1 from an infected source, by sexual act. Table G. Sensitivity analyses 3–5. Simulated sexual activity and HIV-1 transmission events after initiation of ART, for the full week 0 to 24 period, in the three treatment arms corresponding to the Single, Spring-2, and Flamingo trials parametrized according to the sexual risk behavior questionnaire in MSM recruited in the START trial. Table H. Sensitivity analyses 6–8. Simulated sexual activity and HIV-1 transmission events after initiation of ART, for the full week 0 to 24 period, in the three treatment arms corresponding to the Single, Spring-2, and Flamingo trials parametrized according to the sexual risk behavior questionnaire in MSM recruited in the START trial. Figure A. Simulation Flow. Figure B. STATA results for SINGLE <10,000 copies/mL. Figure C. EFV treated patient. Simulated Log10 HIV-RNA over 24 weeks SINGLE <10,000 copies/mL (MEAN ± 95%CI). Figure D. DTG treated patient. Simulated Log10 HIV-RNA over 24 weeks SINGLE <10,000 copies/mL (MEAN ± 95%CI). Figure E. STATA results for SINGLE ≥ 10,000 to <100,000 copies/mL. Figure F. EFV treated patient. Simulated Log10 HIV-RNA over 24 weeks SINGLE ≥ 10,000 to <100,000 copies/mL (MEAN ± 95%CI). Figure G. DTG treated patient. Simulated Log10 HIV-RNA over 24 weeks SINGLE ≥ 10,000 to <100,000 copies/mL (MEAN ± 95%CI). Figure H. STATA results for SINGLE ≥ 100,000 copies/mL. Figure I. EFV treated patient. Simulated Log10 HIV-RNA over 24-weeks SINGLE ≥ 100, [file pone.0219802.s001.docx]

**Technical Appendix:**

**Mathematical modeling of HIV-1 transmission risk from condomless anal intercourse in HIV-infected MSM by the type of initial ART**

## Introduction

We developed a mathematical model to estimate the probability of sexual transmission of HIV among MSM in a serodiscordant dyad over the infected patient’s initial period of HIV antiretroviral therapy (ART). This likelihood depends on the numbers of partners, the number of condomless sex acts and parameters defining HIV-1-infectivity of the infected partner (viral load, type of sexual act, and presence of other sexually transmitted infections). This technical appendix explains the model details, the model parameters and the model outputs.

## Model description

A Discrete Event Simulation (DES) model was developed using Microsoft Excel (2016) and Visual Basic for applications (VBA) to determine the number of transmitted HIV-1 infections after initiation of antiretroviral therapy (ART).

DES is a modeling approach that allows the user to describe the behavior of the individual within a complex system as an ordered sequence of events (which may or may not happen) at a specific point in time and which involves a downstream change of state as the outcome of interest.

Five million theoretical individuals were modeled to determine the number of secondary sexually transmitted HIV-1 infections arising from people initiating dolutegravir [DTG]-containing ART regimens versus infections arising from individuals starting ART containing comparator regimens (efavirenz [EFV], raltegravir [RAL], or darunavir/ritonavir [DRVr]-based ART) and versus no treatment.

The data source for virologic decay of the theoretical patients initiating first-line ART were the SINGLE trial ^(1)^: EFV vs. DTG; SPRING-2: RAL vs. DTG ^(2)^, and FLAMINGO: DRVr vs. DTG ^(3)^. Each patient was progressed iteratively through the model using sexual activity and transmission risk parameters (see below).

The model structure is described in **Figure 1**.

Figure A. Simulation Flow


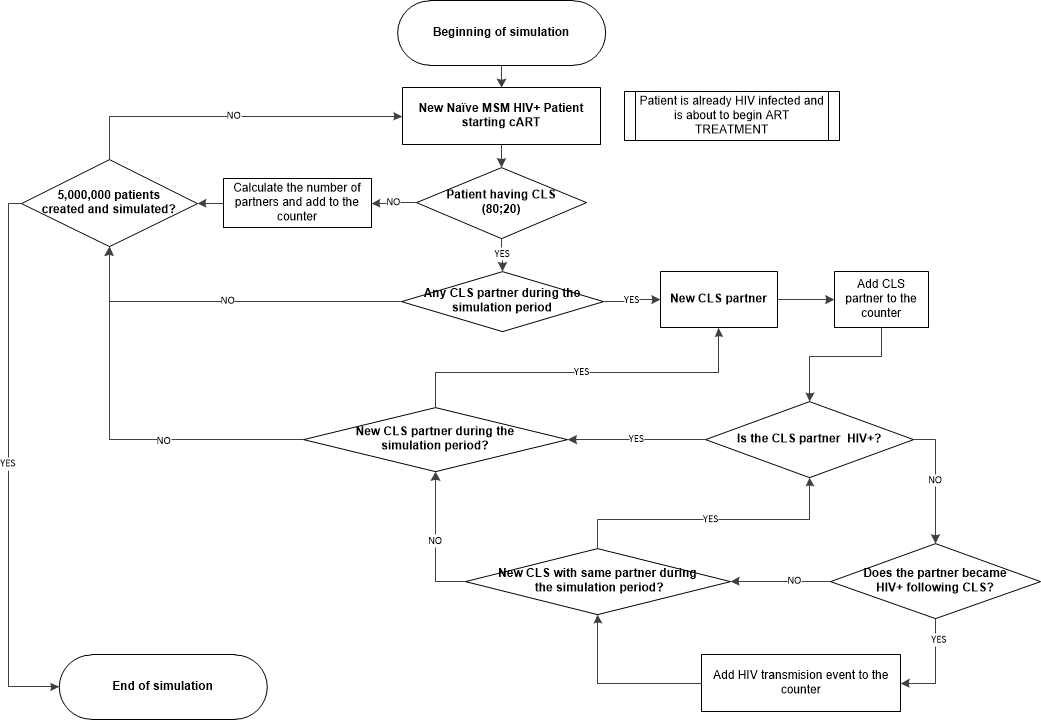


*CLS = Condomless Sex*

At the beginning of the simulation, tracking variables (for time and event counters) are set to 0, and three attributes are fixed for each simulated patient: the number of partners during the period of analysis, sexual relationships per partner and chronology of sexual relationships.

Each patient is cloned to obtain nine identical patients, six of them who will receive treatment based on DTG (SINGLE), DTG (SPRING2), DTG (FLAMINGO), EFV (SINGLE), RAL (SPRING2) and DRVr (FLAMINGO), and another three who will not receive any treatment. For each treatment a viral load decay curve is set at the beginning of the simulation and it has been assumed that patients without treatments remain at the same viral load set point during the simulation.

In the first step of the simulation, it is evaluated whether the patient has CLS with a serodiscordant partner (20%) or not (80%). Having assumed that there is no transmission if there are not CLS, in patients without CLS, the simulation calculates and record the number of partners and goes to the next patient.

For patients having CLS, throughout the simulation, the viral load varies based on the treatment assignment viral load decay curve. Each time that a sexual event occurs in the pre-specified chronology, determination of whether a transmission event occurs is based on the probability of transmission that is determined by the viral load at the time of the event. If the sexual partner becomes infected in the model, this is recorded in a counter variable, and the simulation continues to assess the next partner.

The process is repeated for each partner until the completion of the simulation time horizon, at which point all patient’s data are recorded and the time variable is reset to 0 to continue the simulation with the next simulated patient.

## Model parameters

## Number of partners and number and type of condomless sexual acts

The patient’s sexual behavior (number of partners per patient, frequency and type of relations) has been simulated based on the results of the START study (**Tables 1,2**) ^(4)^.

Table A. Condomless sex with an HIV-1-discordant status partner in men who have sex with men MSM*

| **Sexual activity in past 2 months** | **MSM (n = 2559)**  **n (%)** | **Heterosexual men (n = 803)**  **n (%)** | **Women**  **(n = 1239)**  **n (%)** |
| --- | --- | --- | --- |
| Condomless sex with an HIV-1-discordant status partner (CLS-D) | | | |
| - Yes | 513 (20.0) | 76 (9.5) | 178 (14.4) |
| - No | 2046 (80.0) | 727 (90.5) | 1061 (85.6) |
| Total number of CLS-D partners among those reporting CLS-D (*n* = 767) | | | |
| - 1 | 310 (60.4) | 61 (80.3) | 154 (86.5) |
| - 2 | 98 (19.1) | 9 (11.8) | 12 (6.7) |
| - 3-5 | 54 (10.5) | 3 (4.0) | 4 (2.3) |
| - > 5 | 28 (5.5) | 0 (0) | 0 (0) |
| Not given | 23 (4.5) | 3 (3.9) | 8 (4.5) |
| Frequency of CLS-D† among those reporting CLS-D (*n* = 697‡) | | | |
| - Once | 141 (30.7) | 17 (23.0) | 41 (25.0) |
| - 2–10 times | 217 (47.3) | 42 (56.8) | 90 (54.9) |
| - 11–30 times | 41 (8.9) | 5 (6.8) | 11 (6.7) |
| - > 30 times | 27 (5.9) | 5 (6.8) | 11 (6.7) |
| - Not given | 33 (7.2) | 5 (6.8) | 11 (6.7) |

**Defined based on gender, mode of HIV-1 transmission, and sexual behavior in past 2 months. Men who reported either MSM transmission or anal sex with another man in past 2 months were classified as MSM.*

*†Question not asked of 566 participants who did not receive version 2 of the transmission risk behavior (TRB) questionnaire.*

*‡Number reporting CLS-D/total number, for subgroup who received version 2 of the TRB questionnaire: 459 of 2179 MSM; 74 of 718 heterosexual men; 164 of 1138 women*.

An 8-weeks time horizon base case has been set using partners and condomless sex relationships from the START data.

A 24-week time horizon sensitivity analysis has been done assuming that, from the START data, those patients reporting only a single partner or two partners for the 8 week period, remain with the same number of partners during the 24 week period and for those reporting 3 or more partners, 1/3 stayed with the same number of partners, another 1/3 doubled the number of partners and the last 1/3 tripled the number of partners.

An additional 24-wees time horizon sensitivity analysis assumed the same number of partners as the START study participants. In this analysis, we assume that the 3-fold increase in duration can be extrapolated by multiplying by three the number of risky sexual encounters.

To assess the number of transmissions during the time horizon, the model uses trackers that allow us to count the transmissions that have occurred by the end of weeks 4, 8 and 24.

Table B. Numbers of partners according to type of condomless sex with an HIV-1-discordant status partner (CLS-D) in 459 MSM participants reporting at least one episode of CLS-D

| **Type of CLS-D according to gender / sexuality**  **group** | **Reported type of sex with at least 1 partner**  **[n (%)]** | | **Number of partners reported** | | | | | |
| --- | --- | --- | --- | --- | --- | --- | --- | --- |
|  |  |  | **0** | **1** | **2** | **3–5** | **> 5** | **Not answered** |
| **MSM (n = 459)** | | | | | | | | |
| **Anal sex with men** | | | | | | | | |
| Insertive with ejaculation | 182 (39.7) | | 213 (46.4) | 144 (31.4) | 20 (4.4) | 11 (2.4) | 7  (1.5) | 64 (13.9) |
| Insertive, no ejaculation | 220 (47.9) | | 184 (40.1) | 164 (35.7) | 27 (5.9) | 14 (3.1) | 15 (3.3) | 55 (12.0) |
| Receptive | 345 (75.2) | | 79 (17.2) | 243 (52.9) | 61 (13.3) | 18 (3.9) | 23 (5.0) | 35 (7.6) |
| **Vaginal sex with women** | | | | | | | | |
| Insertive with ejaculation | 22 (4.8) | | 57 (12.4) | 12  (2.6) | 5  (1.1) | 4  (0.9) | 1 (0.2) | 380 (82.8) |
| Insertive, no ejaculation | 32 (7.0) | | 47 (10.2) | 21  (4.6) | 5  (1.1) | 5  (1.1) | 1 (0.2) | 380 (82.8) |
| **Anal sex with women** | | | | | | | | |
| Insertive with ejaculation | 26 (5.7) | | 58 (12.6) | 13  (2.8) | 5  (1.1) | 6  (1.3) | 2 (0.4) | 375 (81.7) |
| Insertive, no ejaculation | 34 (7.4) | | 48 (10.5) | 21  (4.6) | 8  (1.7) | 2  (0.4) | 3 (0.7) | 377 (82.1) |
| **Heterosexual men (n = 74)** | | | | | | | | |
| **Vaginal sex with women** | | | | | | | | |
| Insertive with ejaculation | | 57 (77.0) | 16 (21.6) | 48 (64.9) | 6  (8.1) | 3  (4.1) | 0  (0) | 1 (1.4) |
| Insertive, no ejaculation | | 26 (35.1) | 37 (50.0) | 23 (31.1) | 1  (1.4) | 1  (1.4) | 1 (1.4) | 11 (14.9) |
| **Anal sex with women** | | | | | | | | |
| Insertive with ejaculation | | 11 (14.9) | 54 (73.0) | 7  (9.5) | 3  (4.1) | 1  (1.4) | 0 (  0) | 9 (12.2) |
| Insertive, no ejaculation | | 12 (16.2) | 52 (70.3) | 8  (10.8) | 4  (5.4) | 0  (0) | 0  (0) | 10 (13.5) |
| **Women (n = 164)** | | | | | | | | |
| Vaginal sex with men | | 157 (95.7) | 3  (1.8) | 142 (86.6) | 12 (7.3) | 3  (1.8) | 0  (0) | 4 (2.4) |
| Anal sex with men | | 18 (11.0) | 124 (75.6) | 16  (9.8) | 2  (1.2) | 0  (0) | 0  (0) | 22 (13.4) |

## Viral Load

## Initial viral load

#### The initial viral load of each simulated patient was randomly generated from the distribution of the viral load data at the screening visit from all the patients participating in the studies SINGLE ^(1)^, SPRING2 ^(2)^ and FLAMINGO ^(3)^.

The patients were stratified by levels of pretreatment viral load in copies/mL (<10,000; ≥10,000 to < 100,000; ≥100,000).

Table C. Viral load on screening (group <10,000 copies/mL)

| **Study** | **Drug** | **N** | **Viral load** | |
| --- | --- | --- | --- | --- |
|  |  |  | **Mean** | **Standard Deviation** |
| SINGLE | DTG | 57 | 6,142 | 2,223 |
|  | EFV | 55 | 4,922 | 2,754 |
|  | Total | 112 | 5,543 | 2,561 |
| SPRING 2 | DTG | 72 | 4,319 | 2,902 |
|  | RAL | 70 | 5,097 | 2,942 |
|  | Total | 142 | 5,004 | 2,758 |
| FLAMINGO | DTG | 49 | 5,074 | 2,628 |
|  | DRVr | 47 | 5,019 | 2,656 |
|  | Total | 96 | 5,047 | 2,628 |

Table D. Viral load on screening (group ≥10,000 to < 100,000 copies/mL)

| **Study** | **Drug** | **N** | **Viral load** | |
| --- | --- | --- | --- | --- |
|  |  |  | **Mean** | **Standard Deviation** |
| SINGLE | DTG | 178 | 41,025 | 24,384 |
|  | EFV | 181 | 42,374 | 25,931 |
|  | Total | 359 | 41,705 | 25,150 |
| SPRING 2 | DTG | 178 | 36,326 | 22,076 |
|  | RAL | 189 | 42,477 | 25,919 |
|  | Total | 367 | 40,323 | 24,823 |
| FLAMINGO | DTG | 117 | 37,352 | 23,910 |
|  | DRVr | 109 | 35,295 | 22,806 |
|  | Total | 226 | 36,359 | 23,355 |

Table E. Viral load on screening (group ≥100,000 copies/mL)

| **Study** | **Drug** | **N** | **Viral load** | |
| --- | --- | --- | --- | --- |
|  |  |  | **Mean** | **Standard Deviation** |
| SINGLE | DTG | 113 | 333,752 | 344,781 |
|  | EFV | 119 | 371,853 | 381,525 |
|  | Total | 232 | 353,295 | 363,808 |
| SPRING 2 | DTG | 100 | 380,535 | 603,424 |
|  | RAL | 99 | 529,352 | 1,152,520 |
|  | Total | 199 | 454,569 | 919,275 |
| FLAMINGO | DTG | 46 | 329,215 | 295,001 |
|  | DRVr | 47 | 319,642 | 374,913 |
|  | Total | 93 | 324,377 | 335,961 |

## Viral load decay over the simulation time

In those simulated patients who do not receive any treatment, the viral load remains constant throughout the simulation period.

To describe the relationship between the viral load and time in those simulated patients who receive treatment, a fractional polynomial regression was used (5-8).

Fractional polynomial regressions are an extension of the polynomial regression with certain restrictions of the fractional powers used:

· Fractional polynomial of one degree: FPI (p) Y = β_0_ + β_1_ x^p^

· Fractional polynomial of two degrees: FPII (p, q) Y = β_0_ + β_1_ x^p^ + β_2_ x^q^

· Power restrictions to [-2, -1, -0.5, 0.0, 0.5, 1, 2, 3]

· Power 0 is interpreted as natural log. Y = β_0_ + Ln(x)

· If p=q; FP2 (p, p) Y = β_0_ + β_1_ x^p^ + β_2_ x^p^ Ln(x)

To stabilize the variance and meet normality assumptions of residuals, viral load measurements from the SINGLE, SPRING2 and FLAMINGO trials were log_10_ transformed.

The variation of the log_10_ transformed viral load over time was described by a regression using a fractional polynomial of one and two degrees. By convention, these are selected from the subset of power, S = (-2, -1, -0.5, 0.0, 0.5, 1, 2, 3) (This subset of fractional polynomials provides rich and flexible shapes of curves for most practical model-fitting purposes).

The fractional polynomial regression for each stratified viral load level and study arm was obtained using STATA 13.

The results of the regressions obtained and the decay curves for each stratified by viral load category (mean value ± 95% CI) are described below (**Figures 2-31**). For each simulated patient, a decay curve has been generated modeled on the parent decay curves of the analogous arm of the appropriate clinical trial, with random effects driven by the distribution of actual participant variability in the clinical trial.

### Viral load decay regressions

Figure B. STATA results for SINGLE <10,000 copies/mL


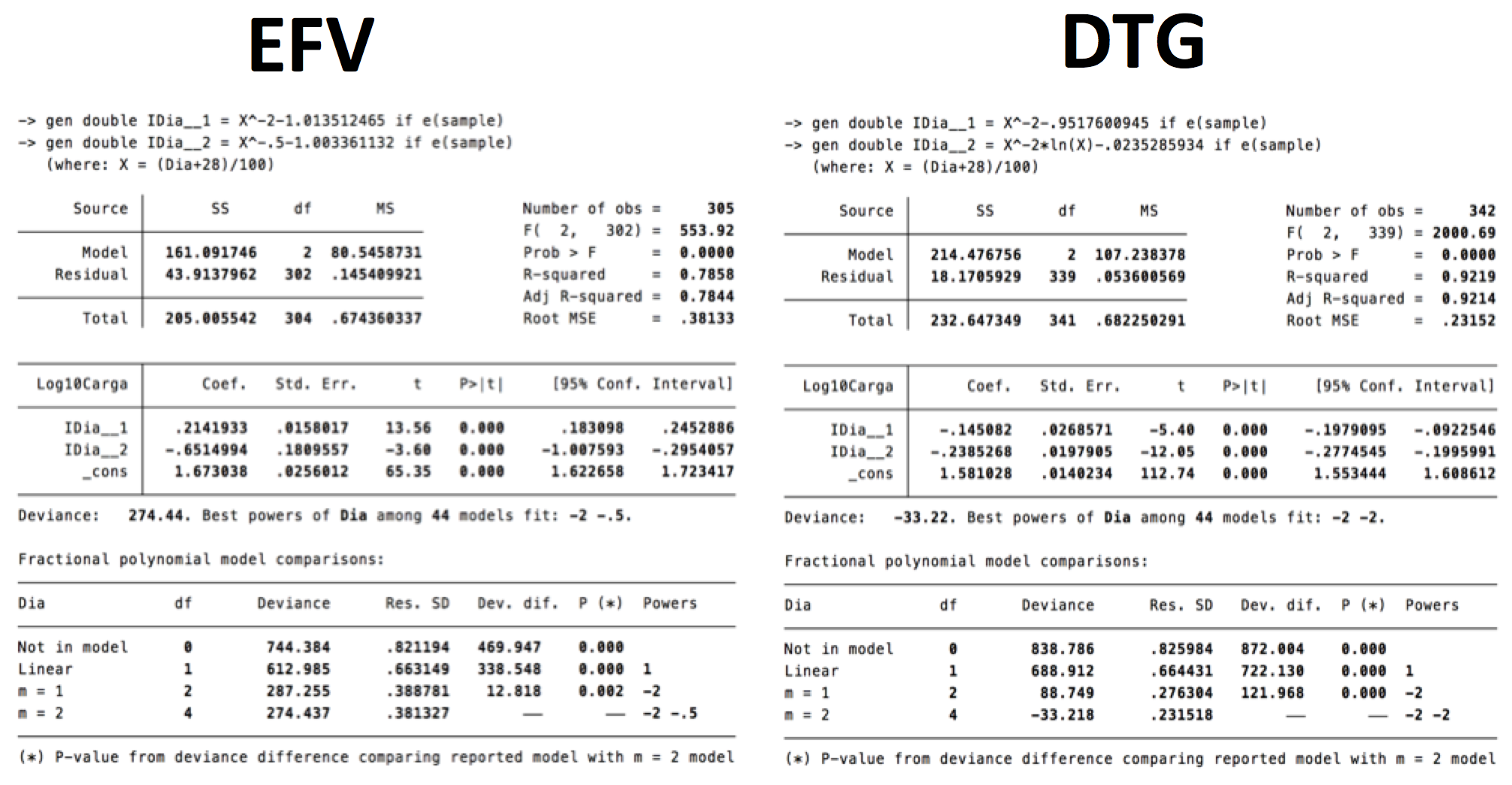


Figure C. EFV treated patient. Simulated Log10 HIV-RNA over 24 weeks SINGLE <10,000 copies/mL (MEAN ± 95%CI)


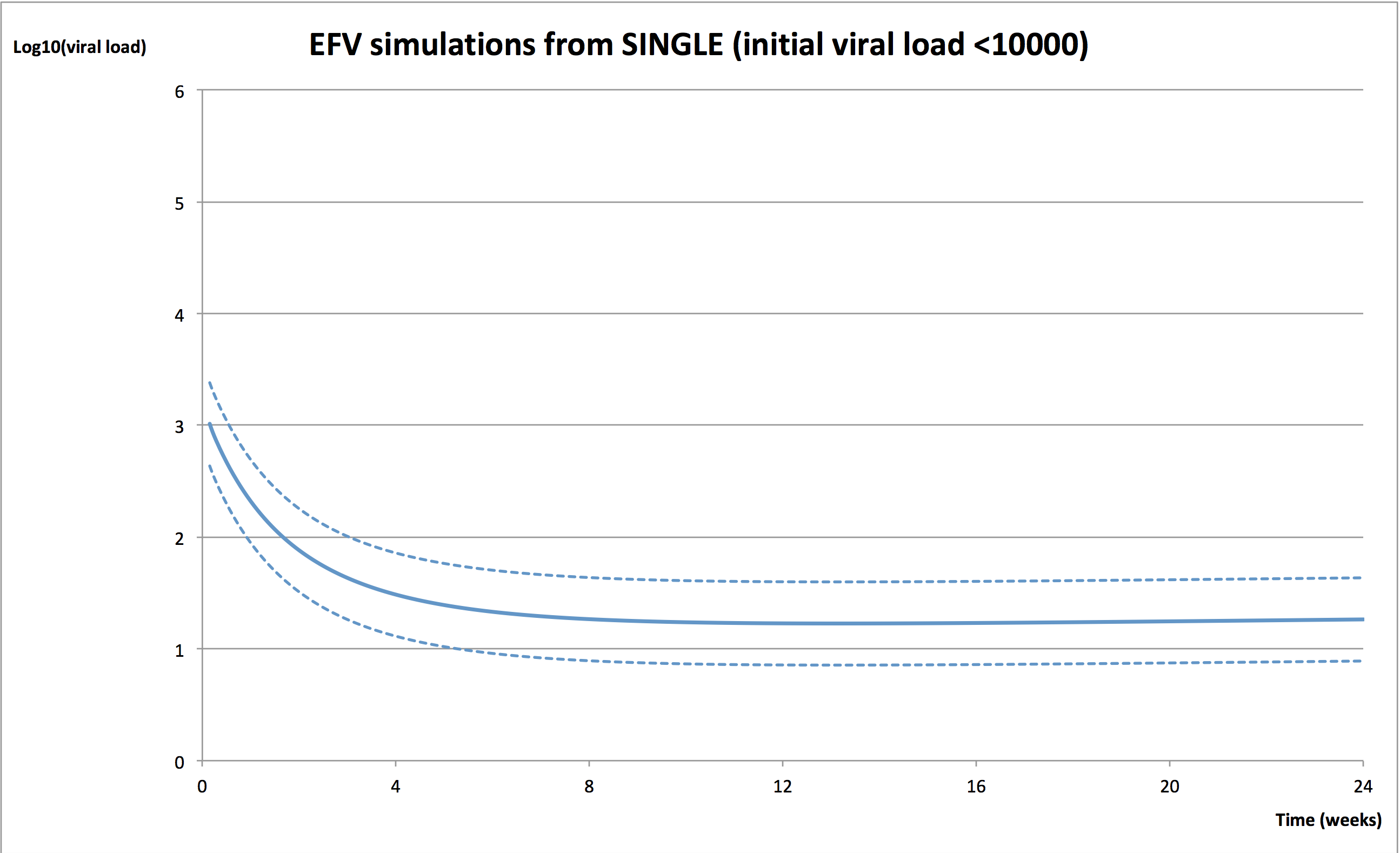


Figure D. DTG treated patient. Simulated Log10 HIV-RNA over 24 weeks SINGLE <10,000 copies/mL (MEAN ± 95%CI)


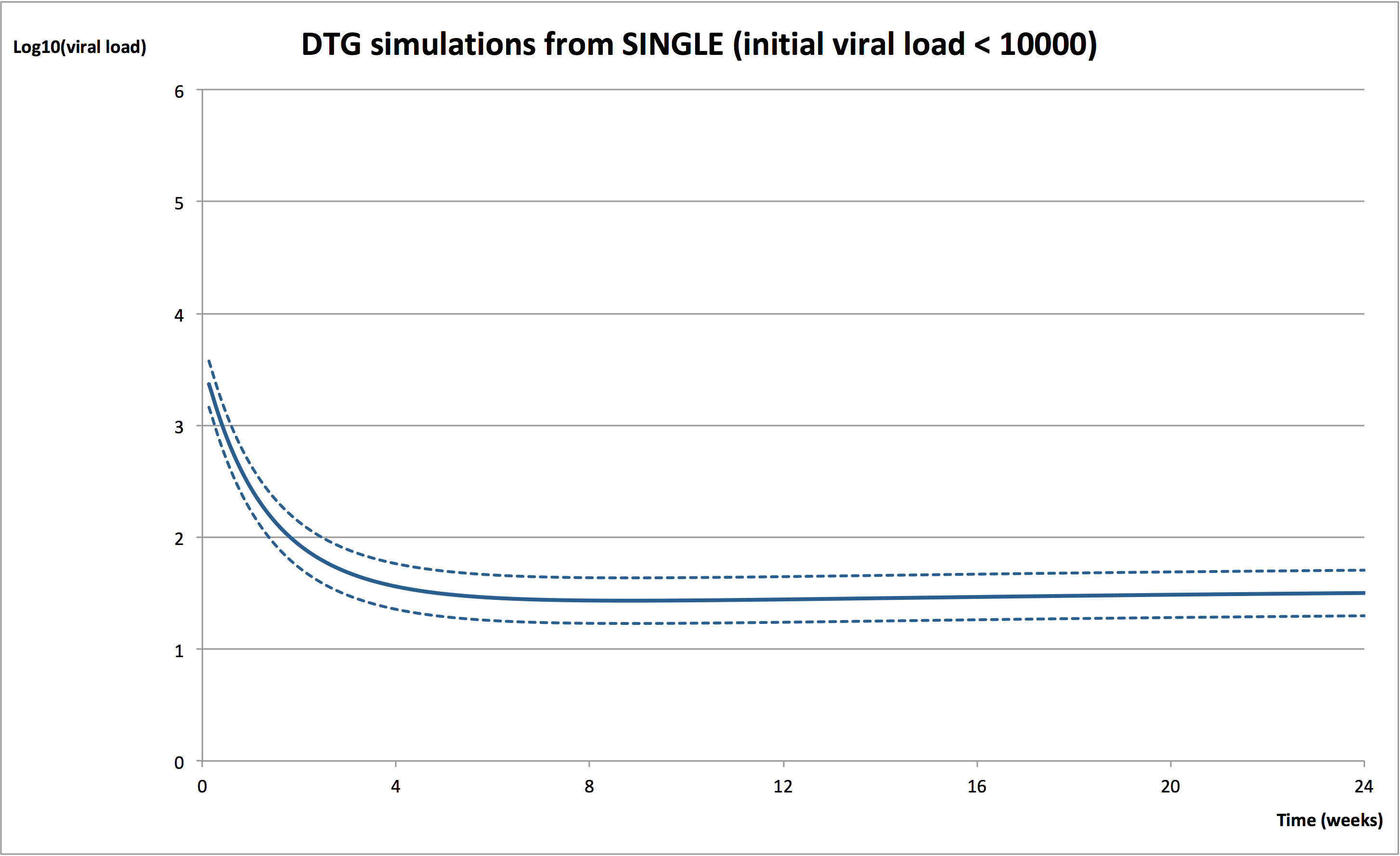


Figure E. STATA results for SINGLE ≥ 10,000 to <100,000 copies/mL


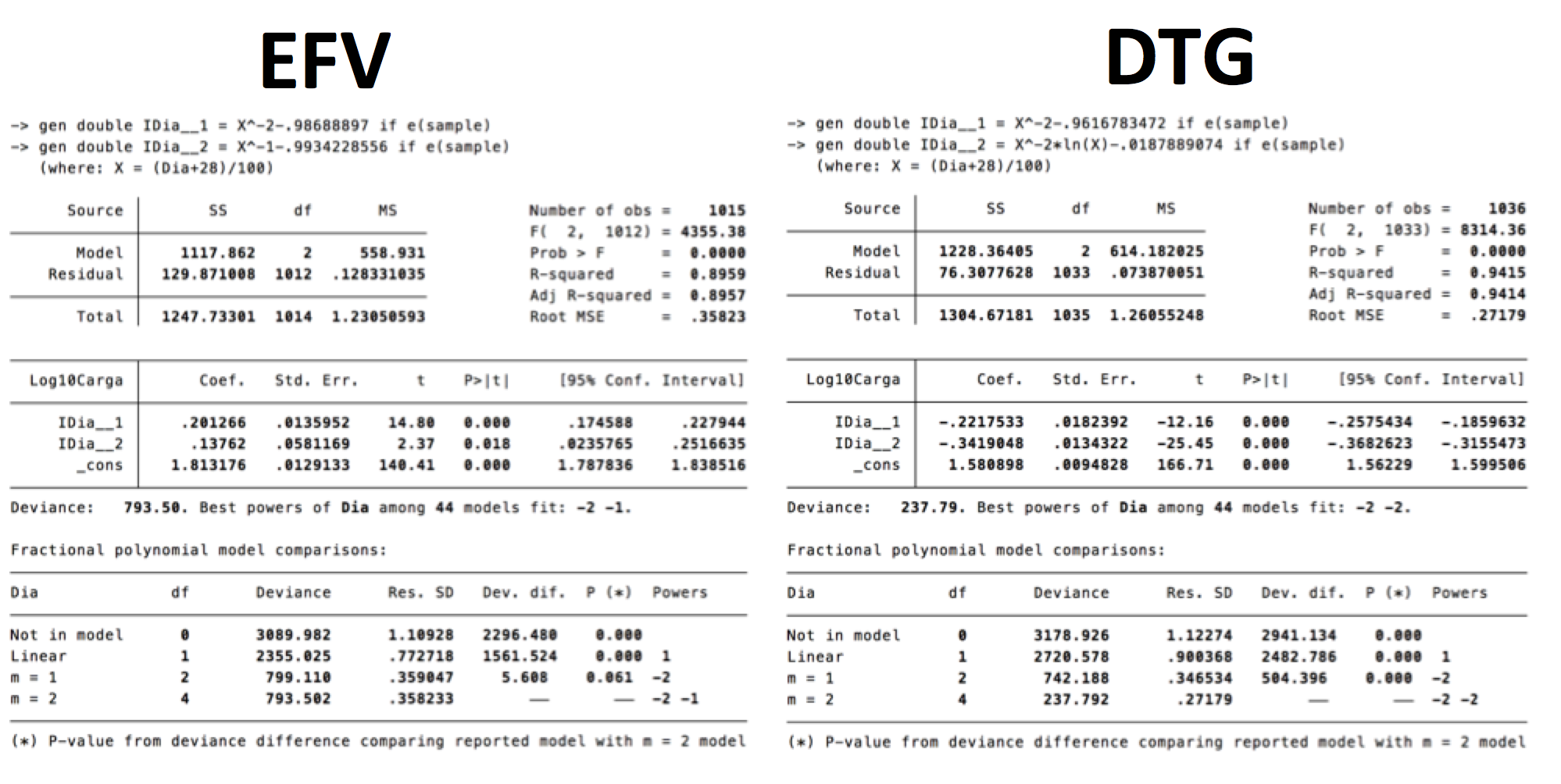


Figure F. EFV treated patient. Simulated Log10 HIV-RNA over 24 weeks SINGLE ≥ 10,000 to <100,000 copies/mL (MEAN ± 95%CI)


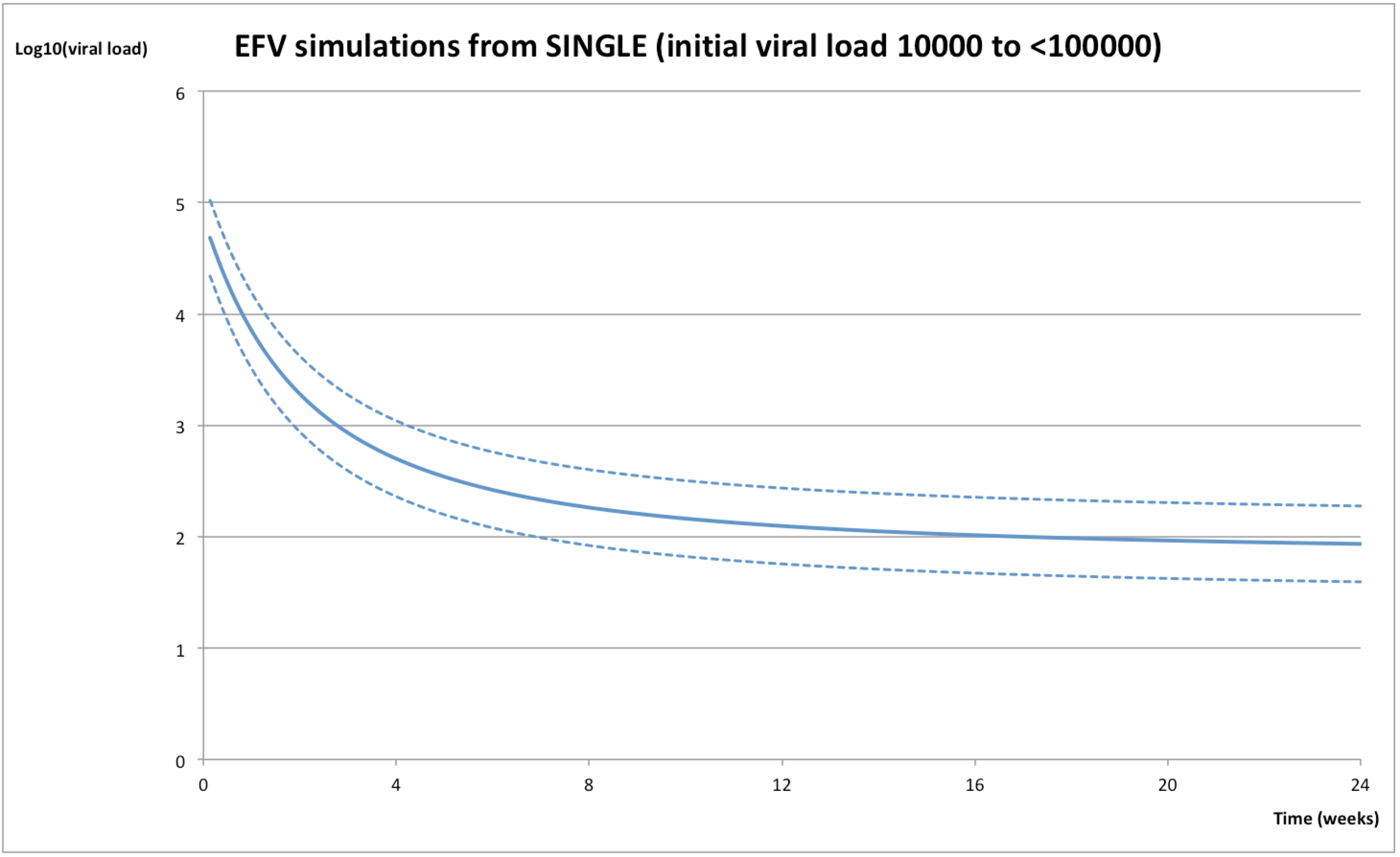


Figure G. DTG treated patient. Simulated Log10 HIV-RNA over 24 weeks SINGLE ≥ 10,000 to <100,000 copies/mL (MEAN ± 95%CI)


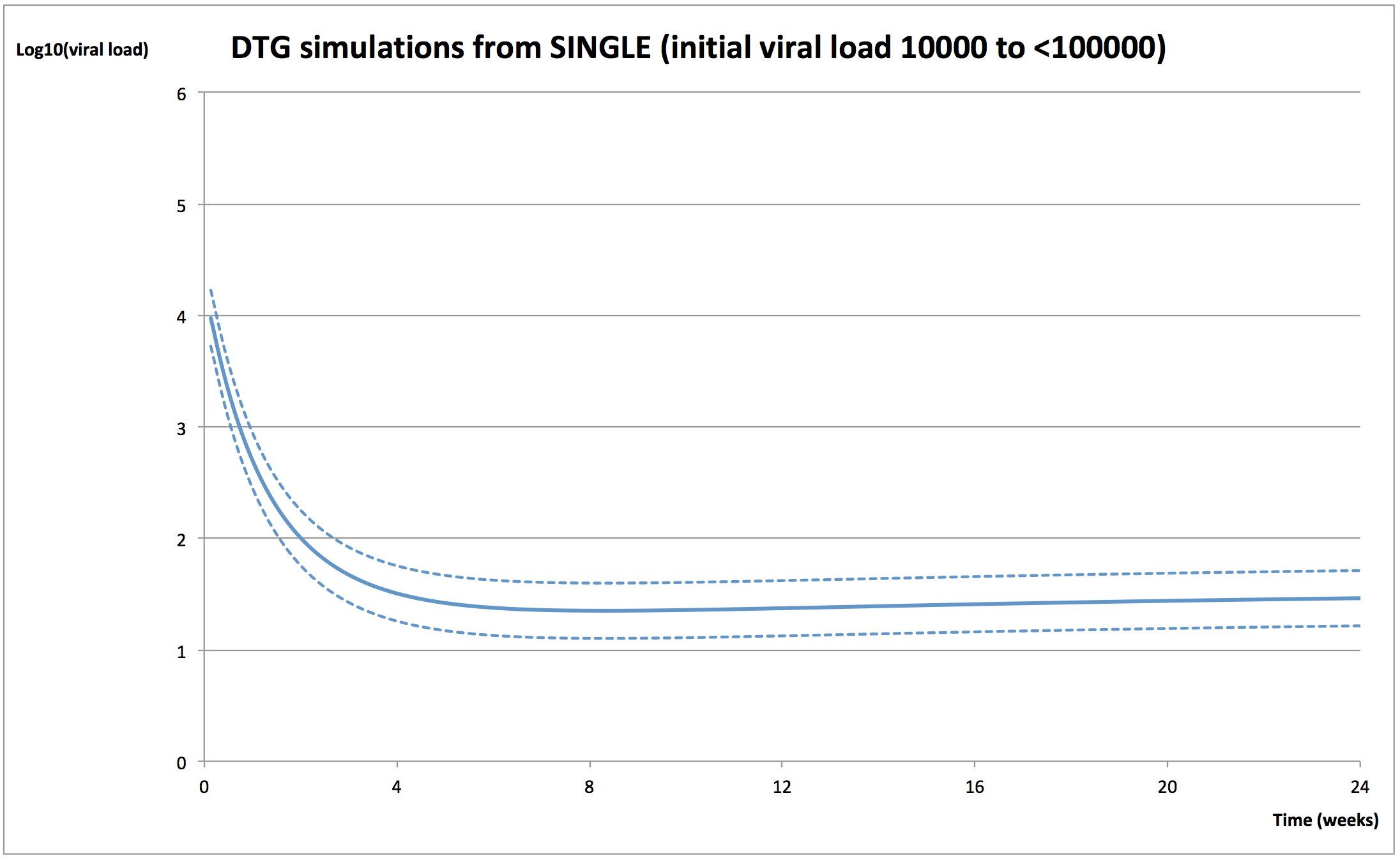


Figure H. STATA results for SINGLE ≥ 100,000 copies/mL


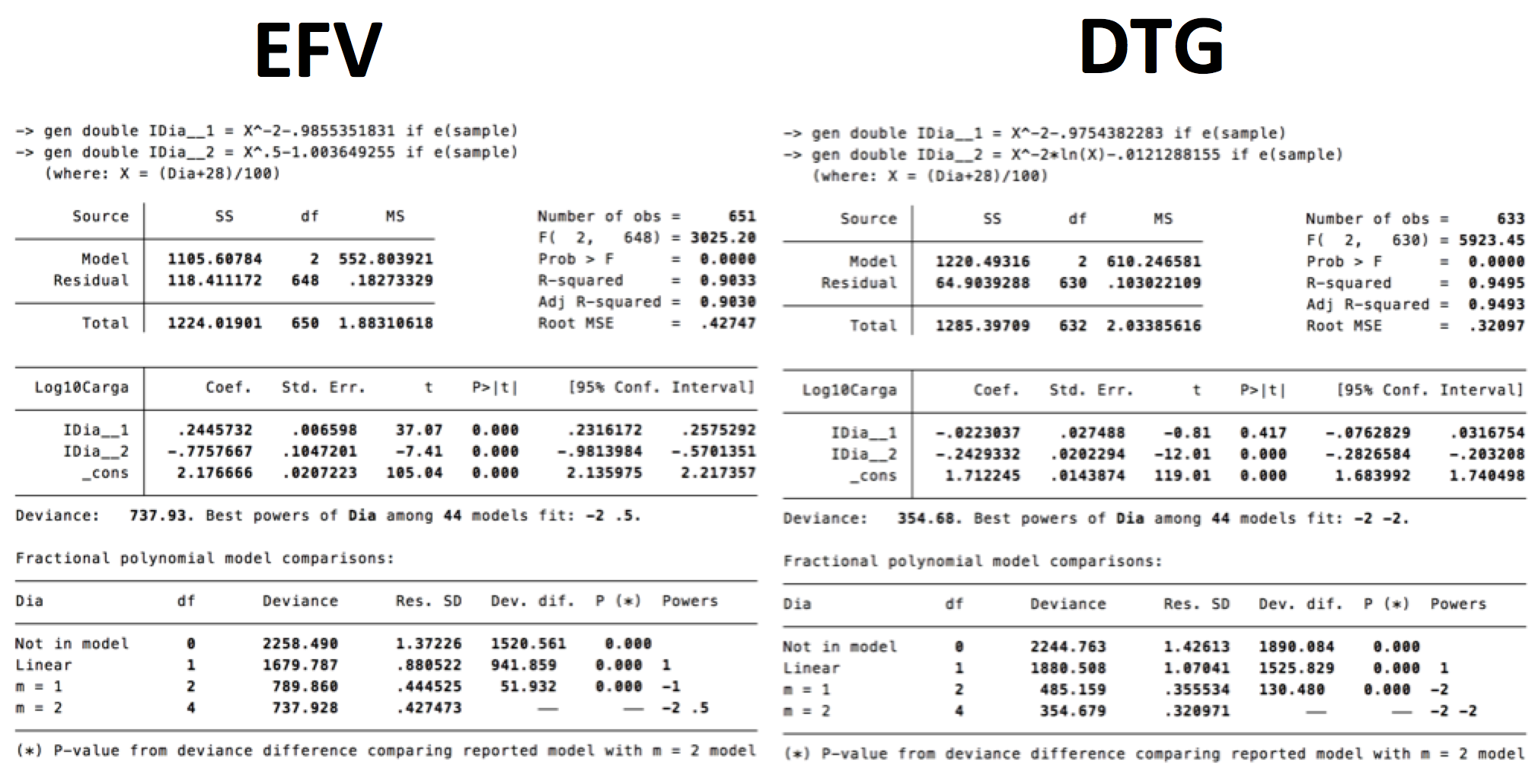


Figure I. EFV treated patient. Simulated Log10 HIV-RNA over 24-weeks SINGLE ≥ 100,000 copies/mL (MEAN ± 95%CI)


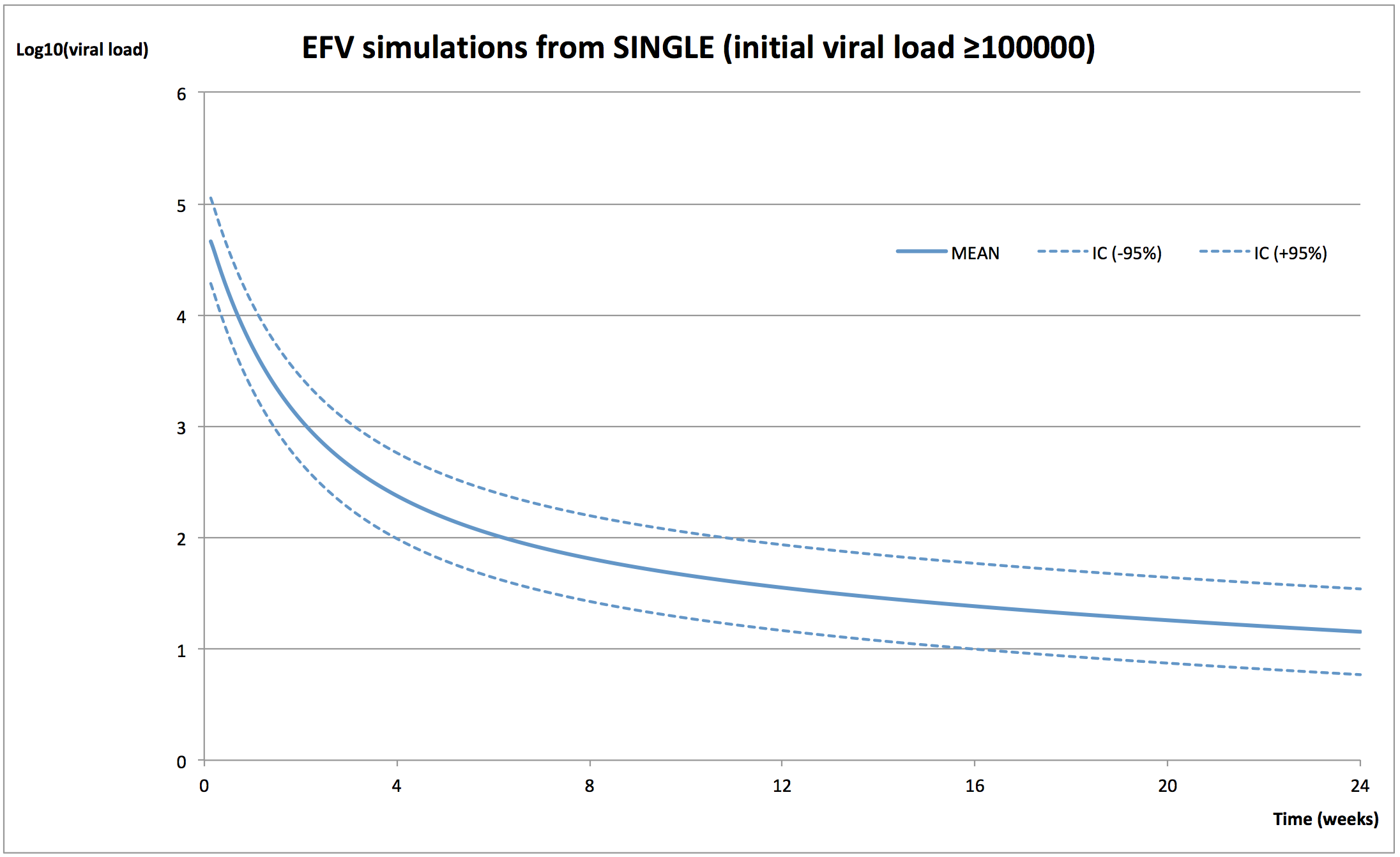


Figure J. DTG treated patient. Simulated Log10 HIV-RNA over 24-weeks SINGLE ≥ 100,000 copies/mL (MEAN ± 95%CI)


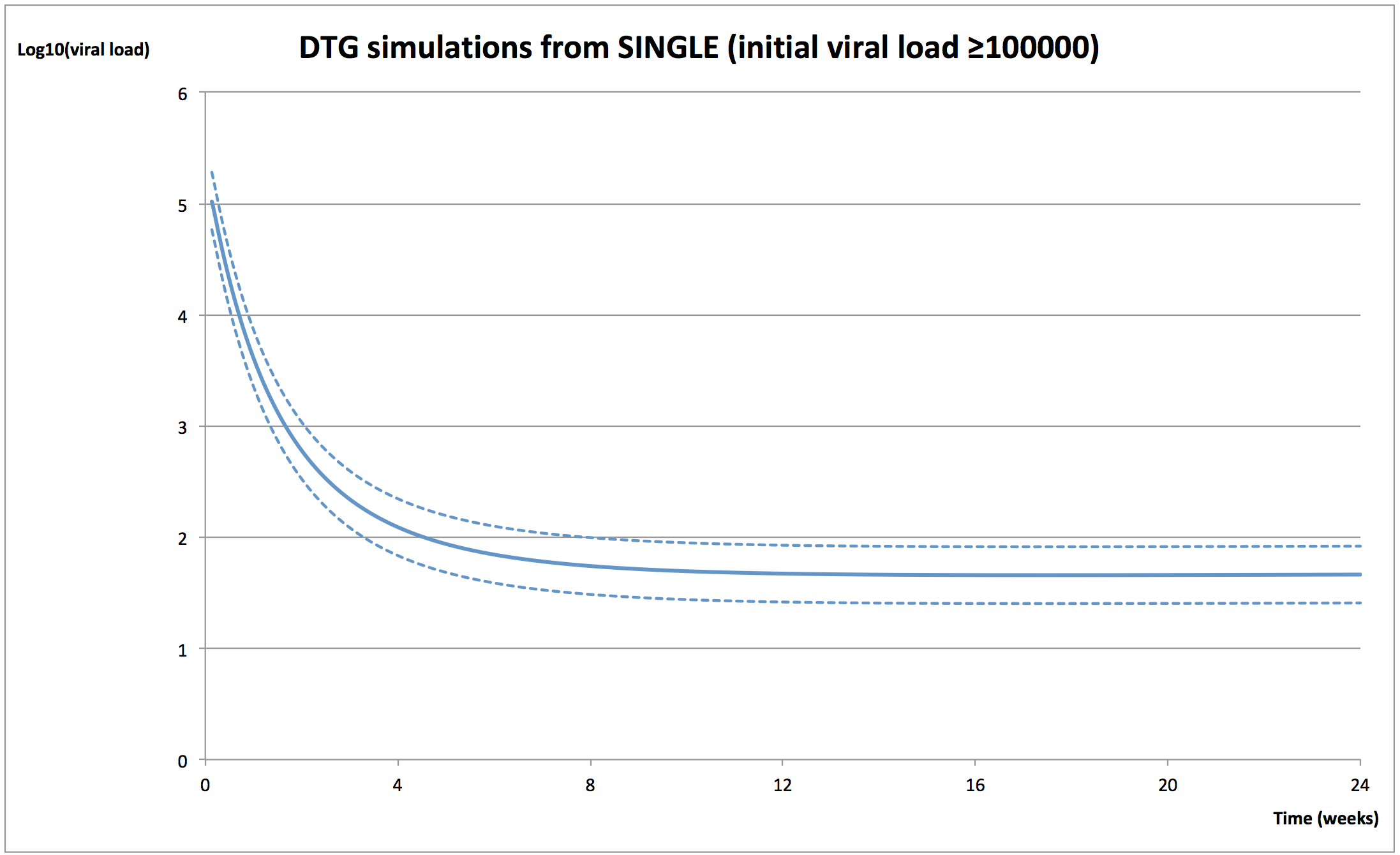


Figure K. STATA results for SPRING2 <10,000 copies/mL


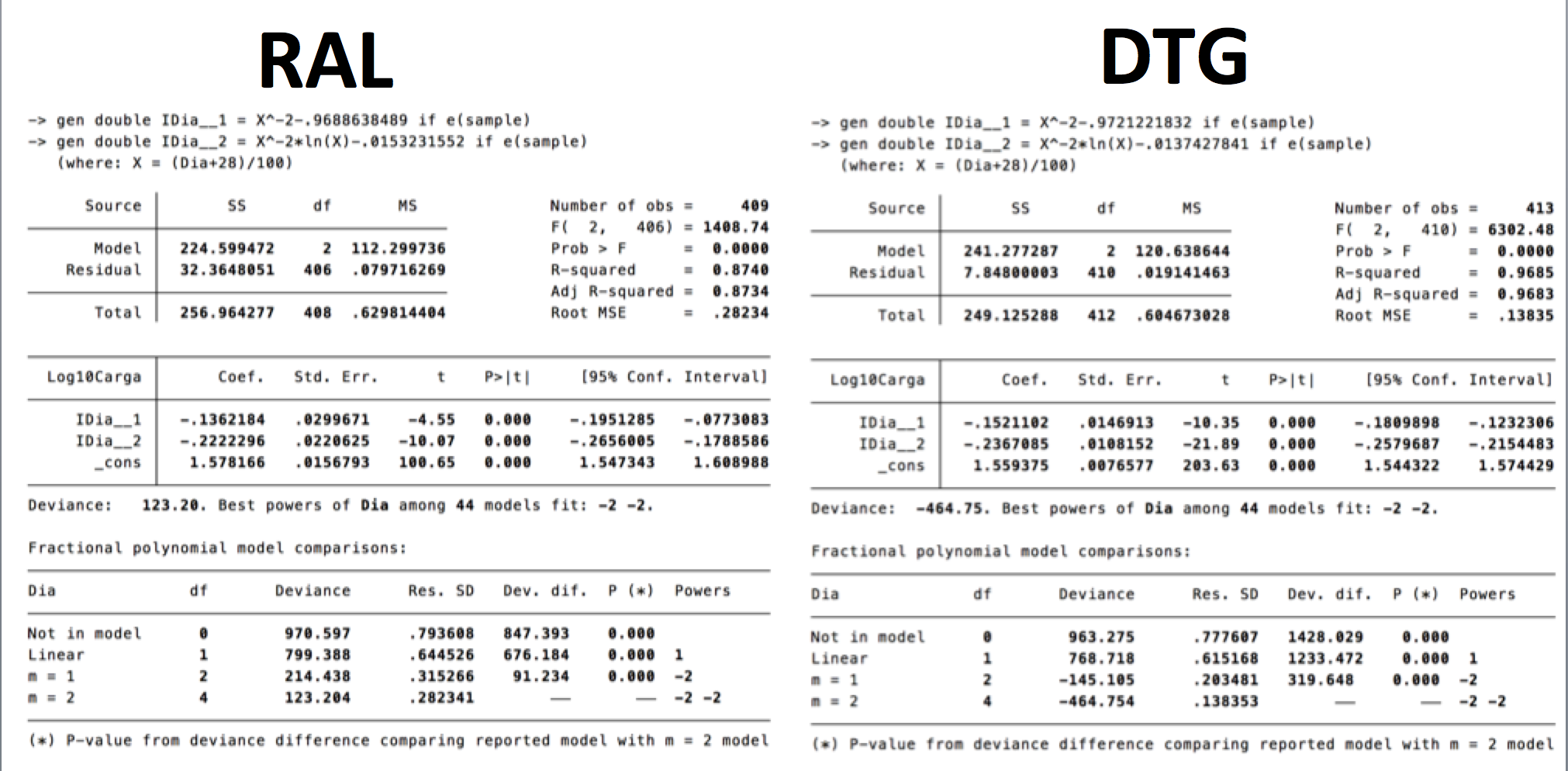


Figure L. RAL treated patient. Simulated Log10 HIV-RNA over 24-weeks SPRING2 <10,000 copies/mL (MEAN ± 95%CI)


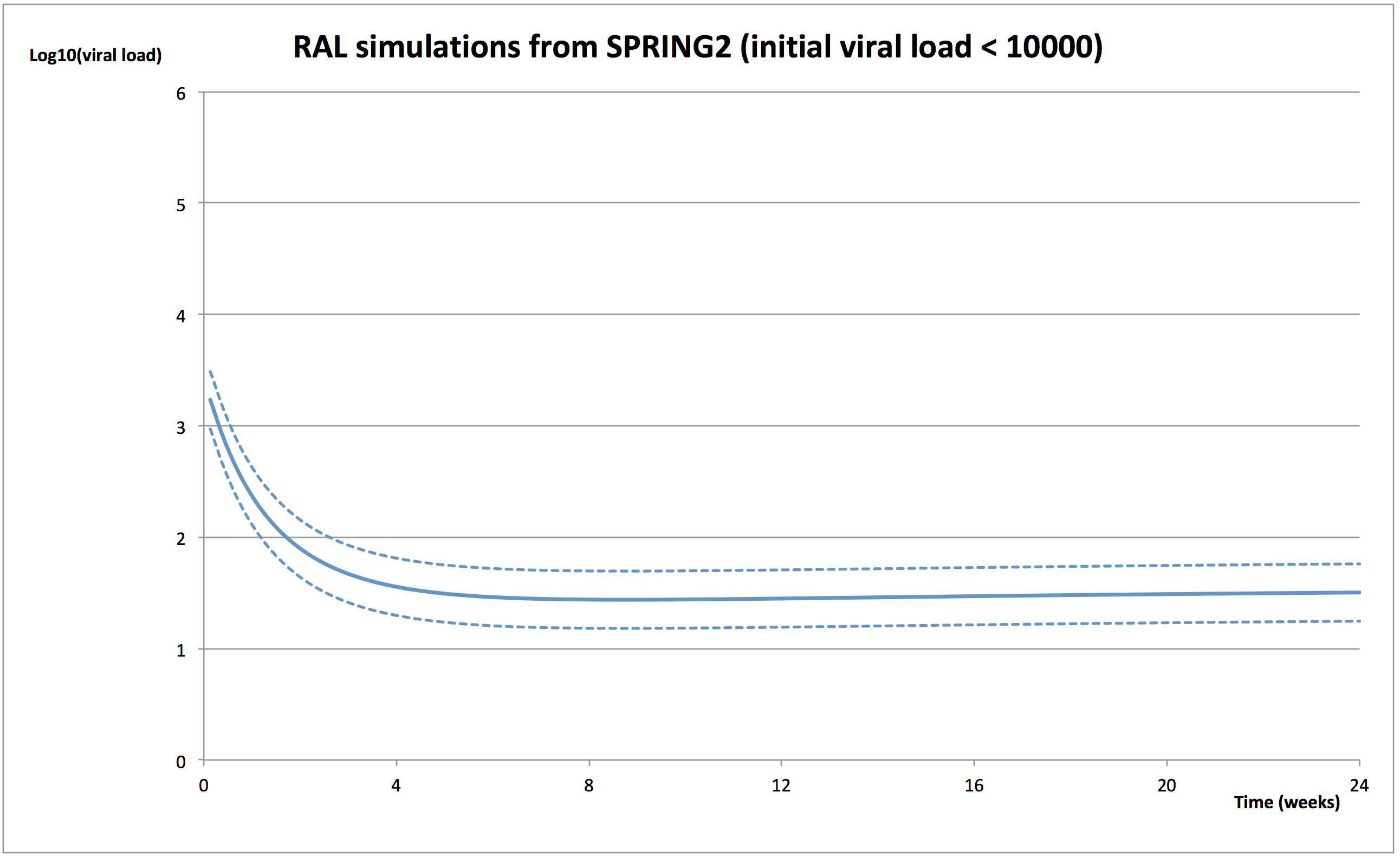


Figure M. DTG treated patient. Simulated Log10 HIV-RNA over 24-weeks SPRING2 <10,000 copies/mL (MEAN ± 95%CI)


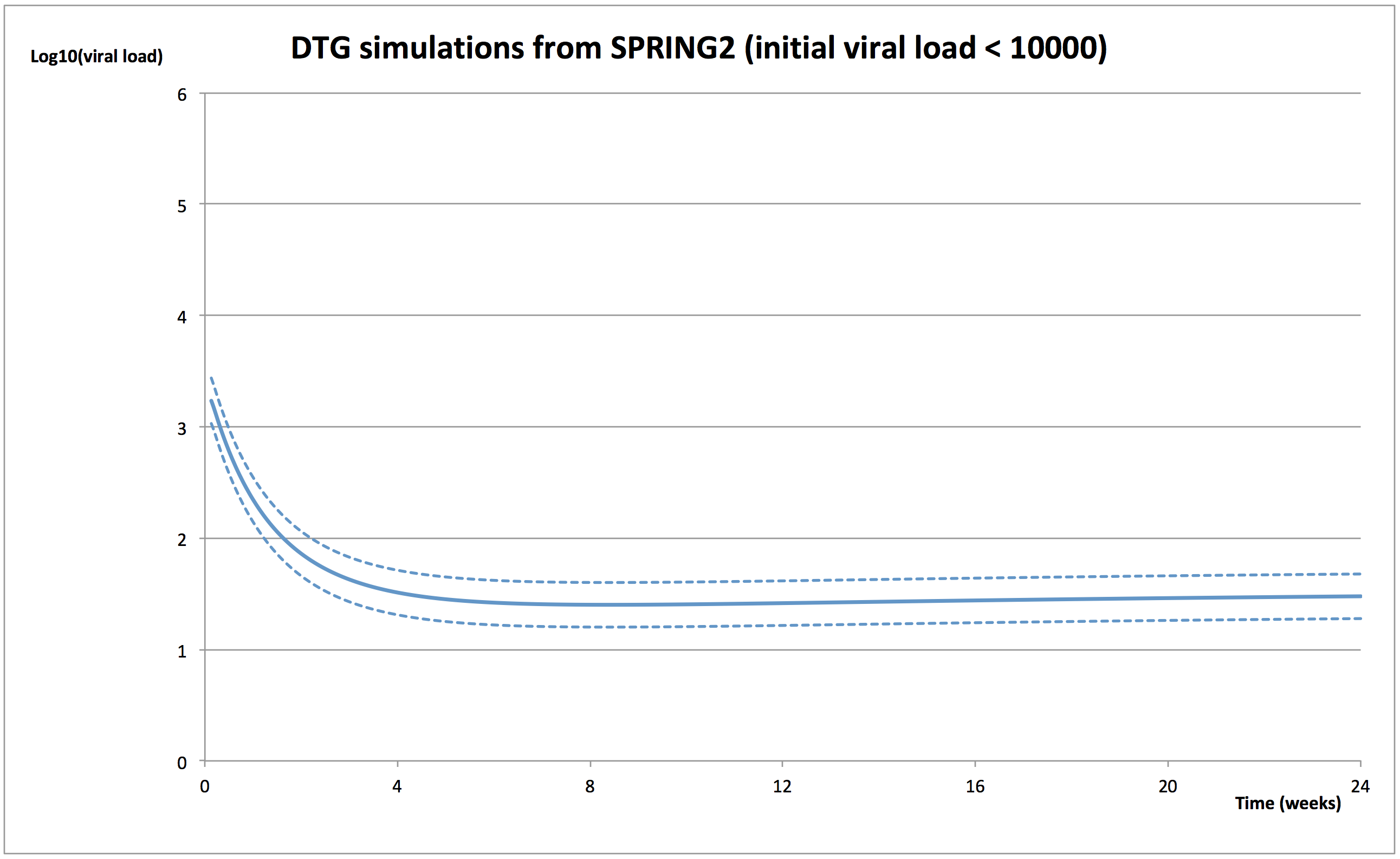


Figure N. STATA results for SPRING2 ≥ 10,000 to <100,000 copies/mL


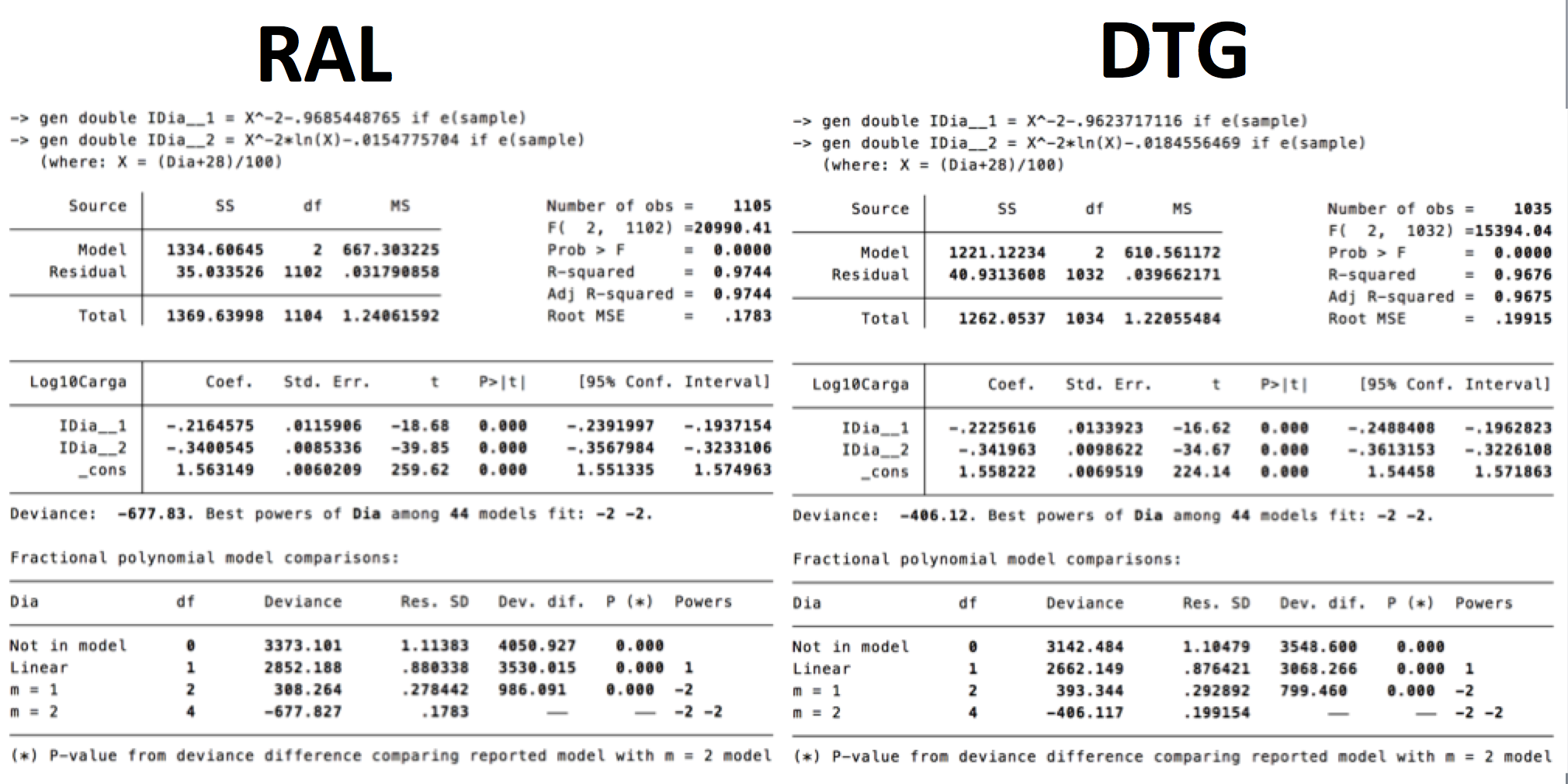


Figure O. RAL treated patient. Simulated Log10 HIV-RNA over 24 weeks SPRING2 ≥ 10,000 to <100,000 copies/mL (MEAN ± 95%CI)


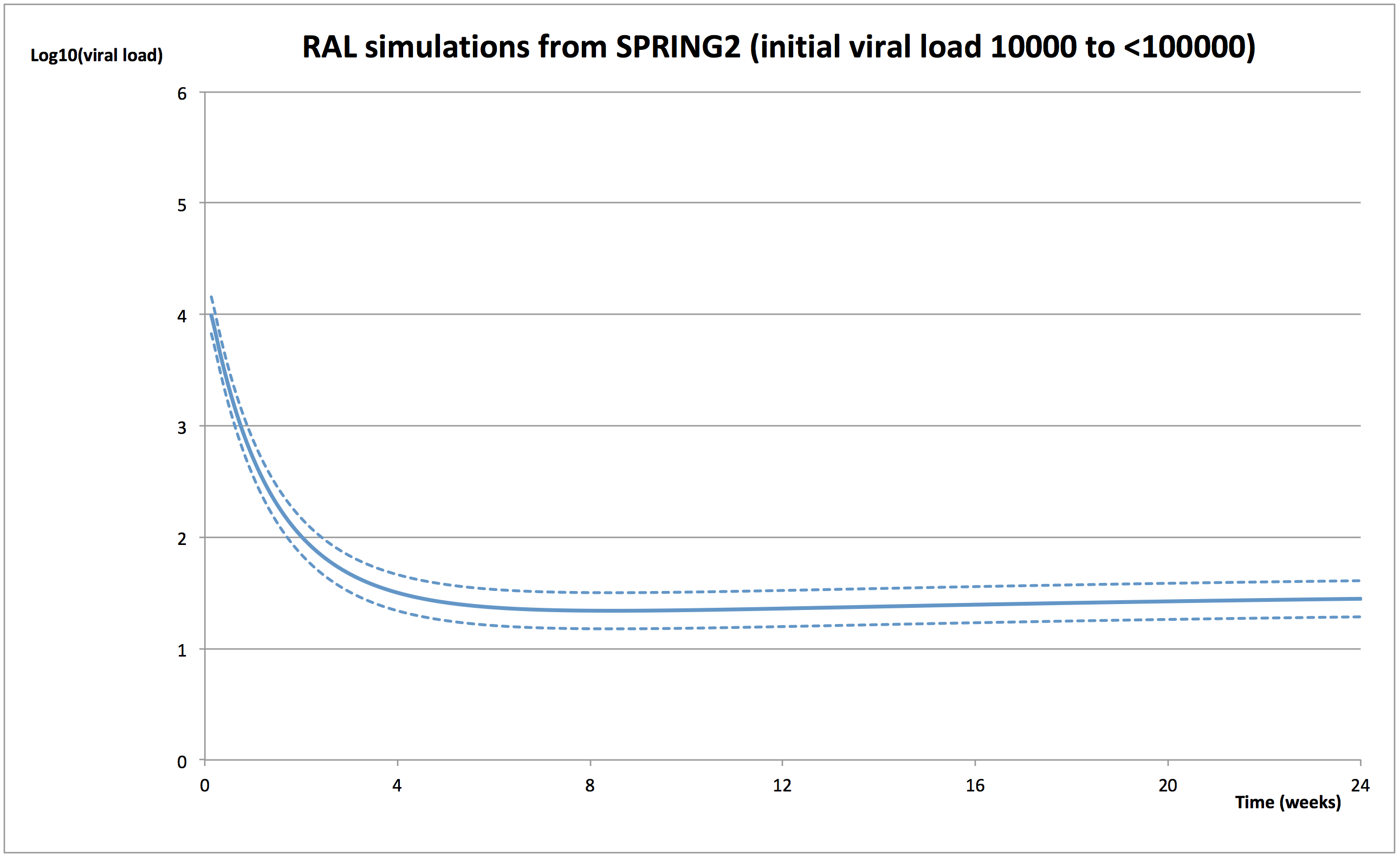


Figure P. DTG treated patient. Simulated Log10 HIV-RNA over 24 weeks SPRING2 ≥ 10,000 to <100,000 copies/mL (MEAN ± 95%CI)


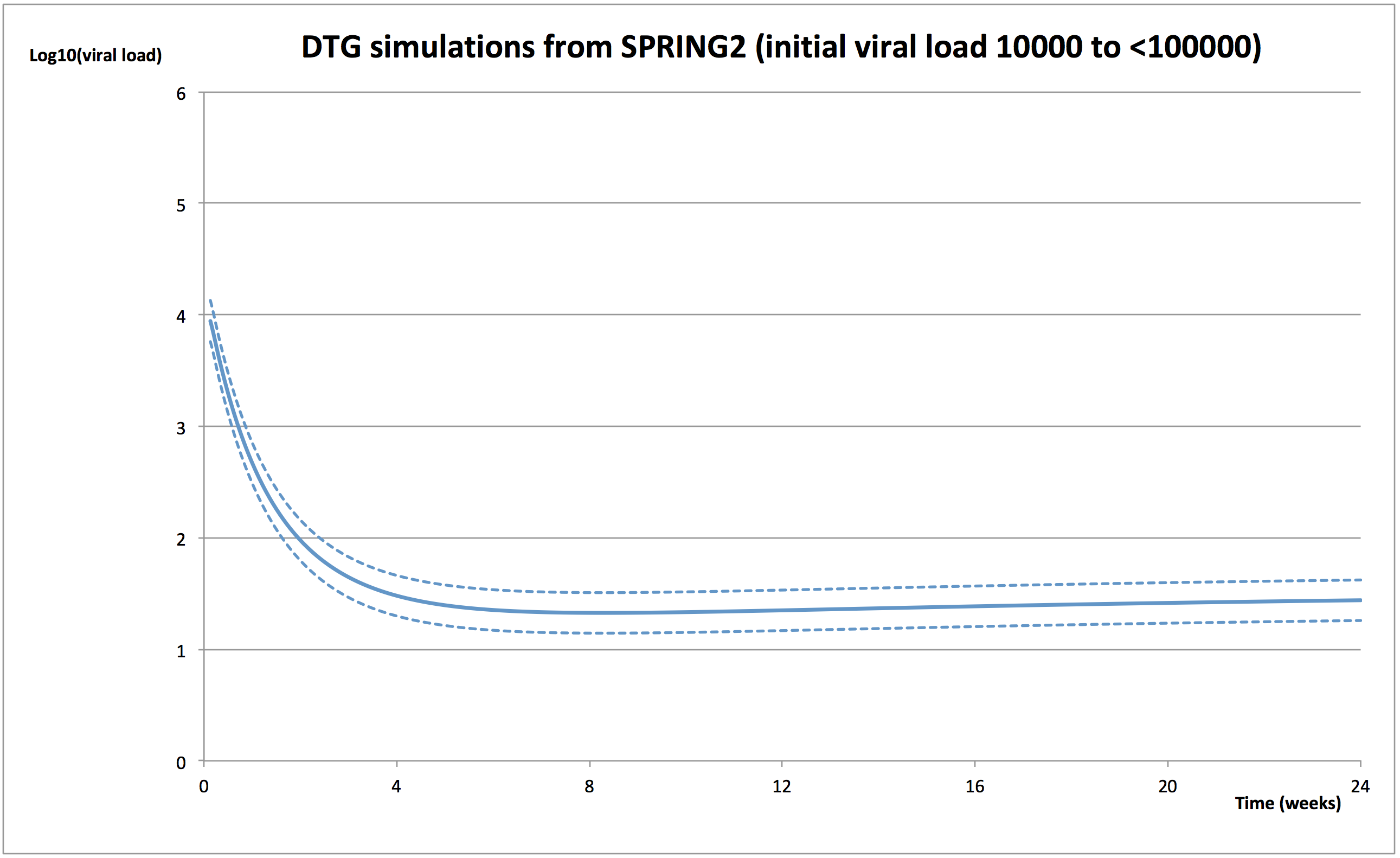


Figure Q. STATA results for SPRING2 ≥ 100,000 copies/mL


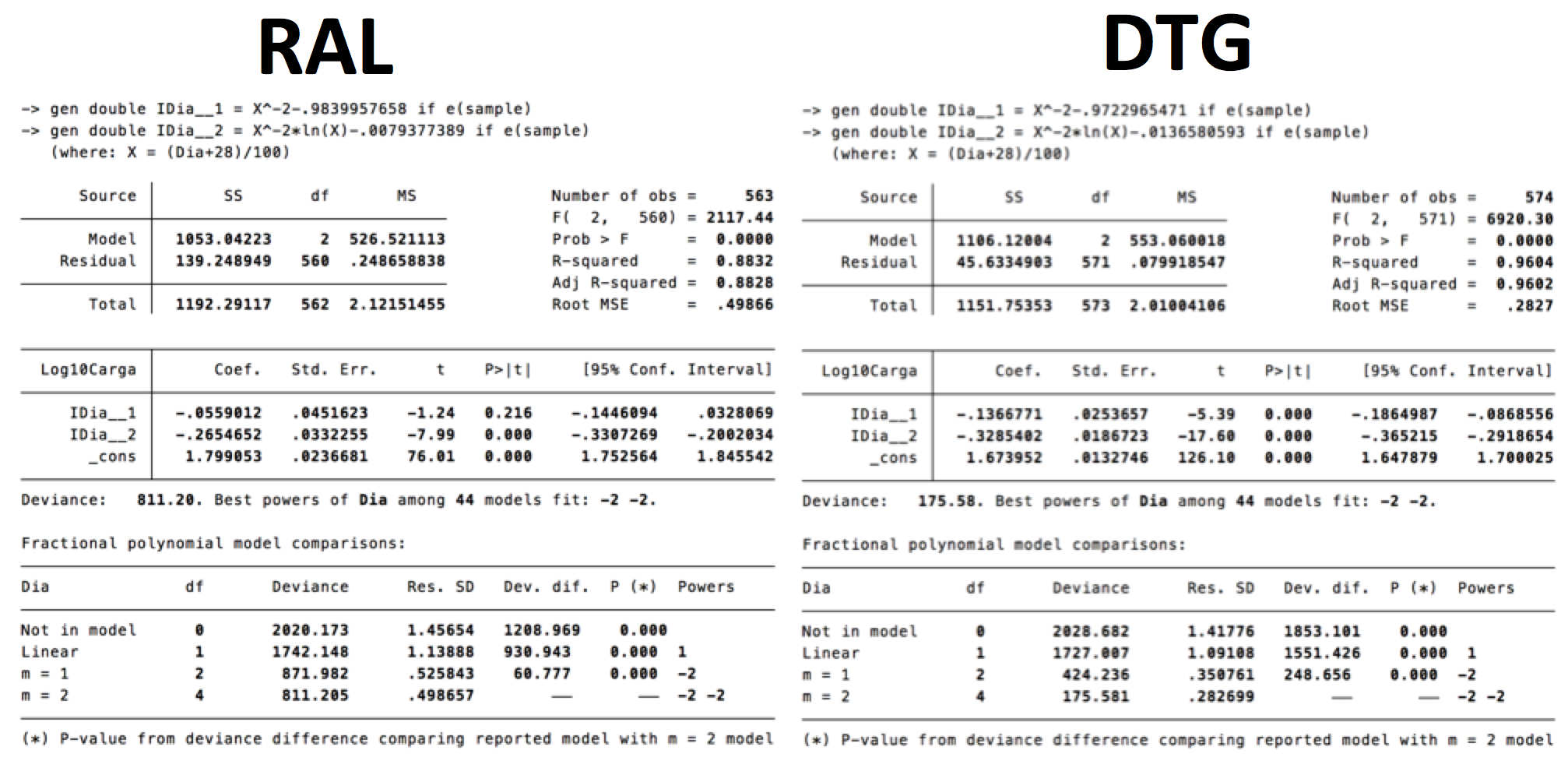


Figure R. RAL treated patient. Simulated Log10 HIV-RNA over 24-weeks SPRING2 ≥ 100,000 copies/mL (MEAN ± 95%CI)


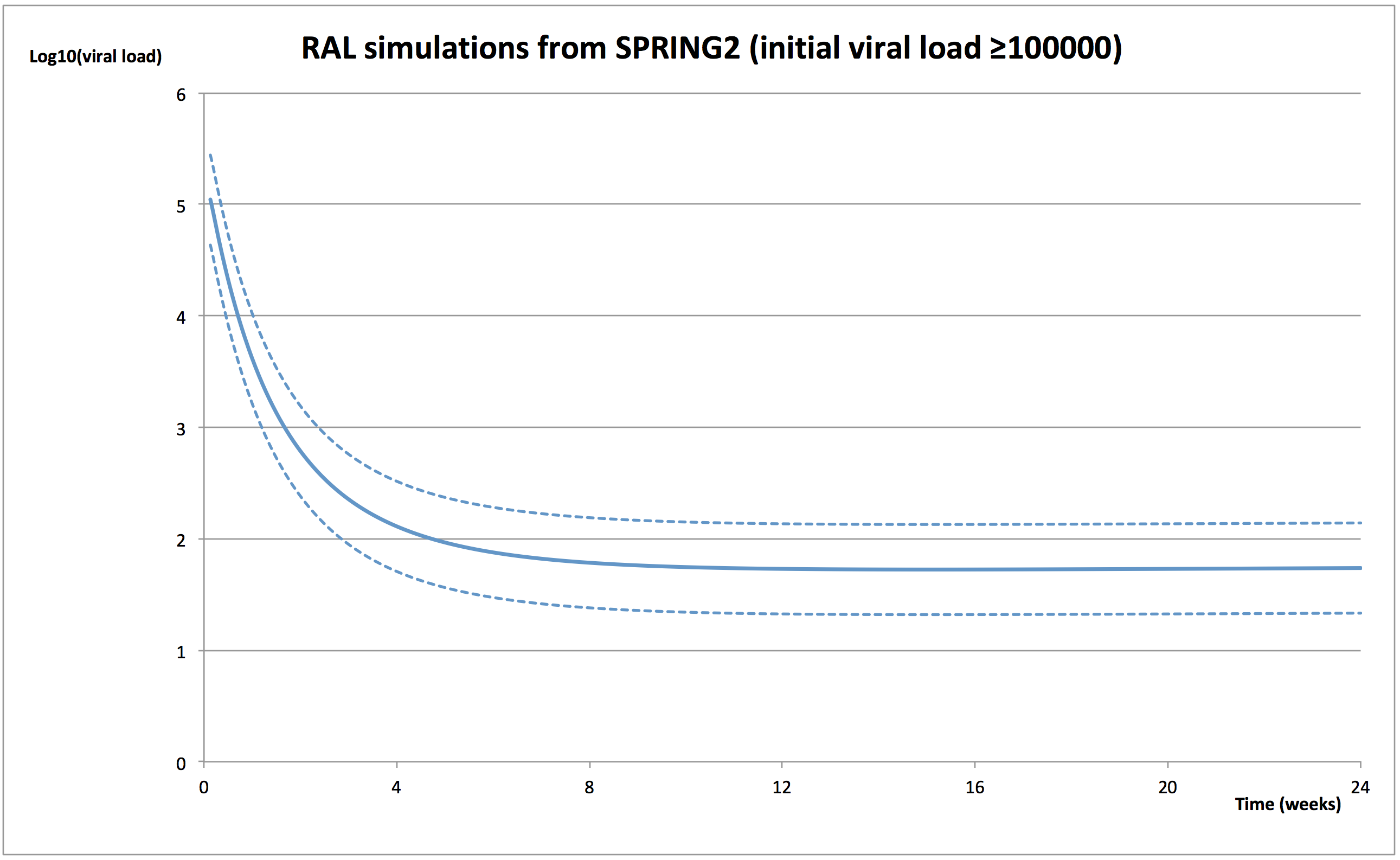


Figure S. DTG treated patient. Simulated Log10 HIV-RNA over 24-weeks SPRING2 ≥ 100,000 copies/mL (MEAN ± 95%CI)


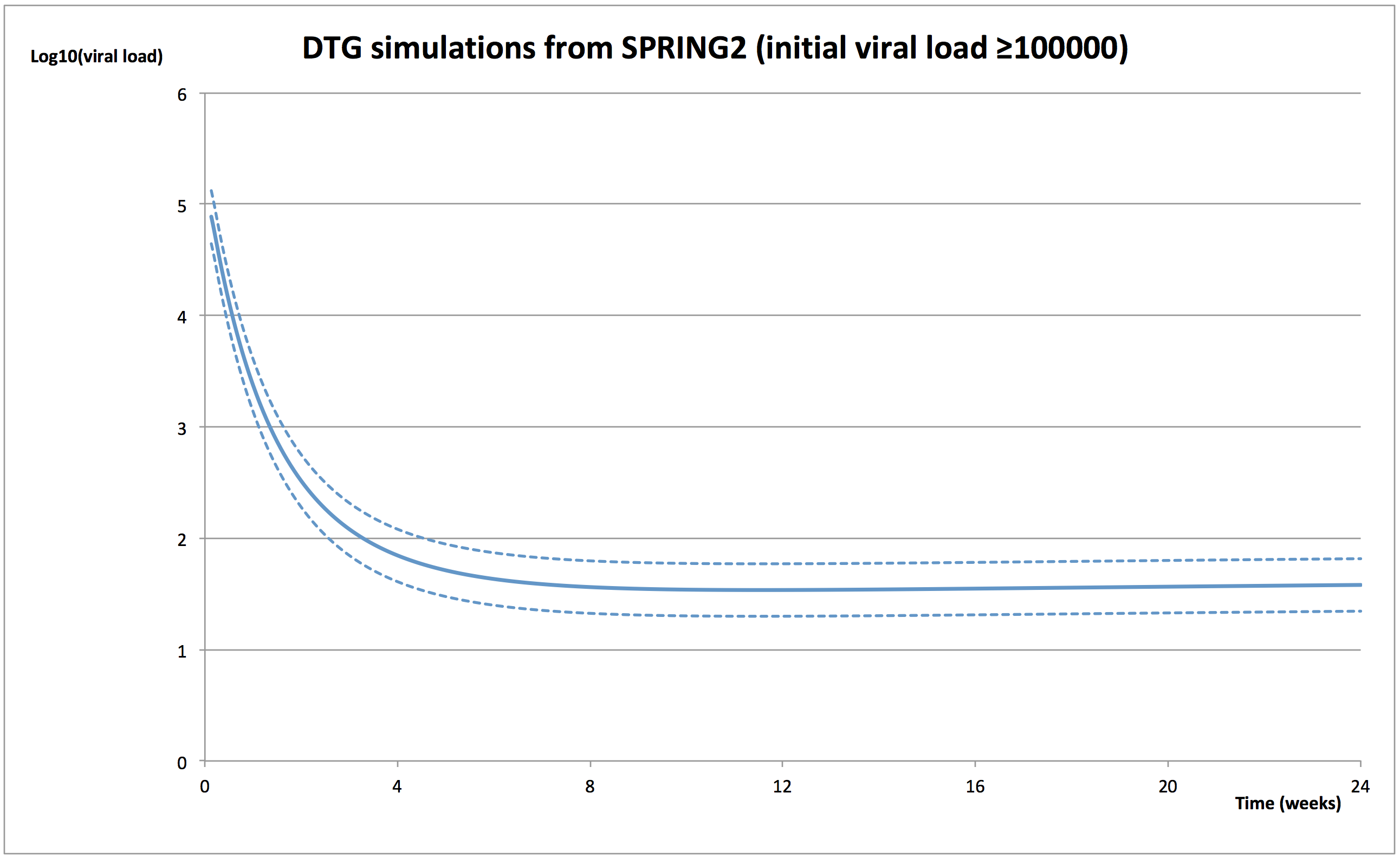


Figure T. STATA results for FLAMINGO < 10,000 copies/mL


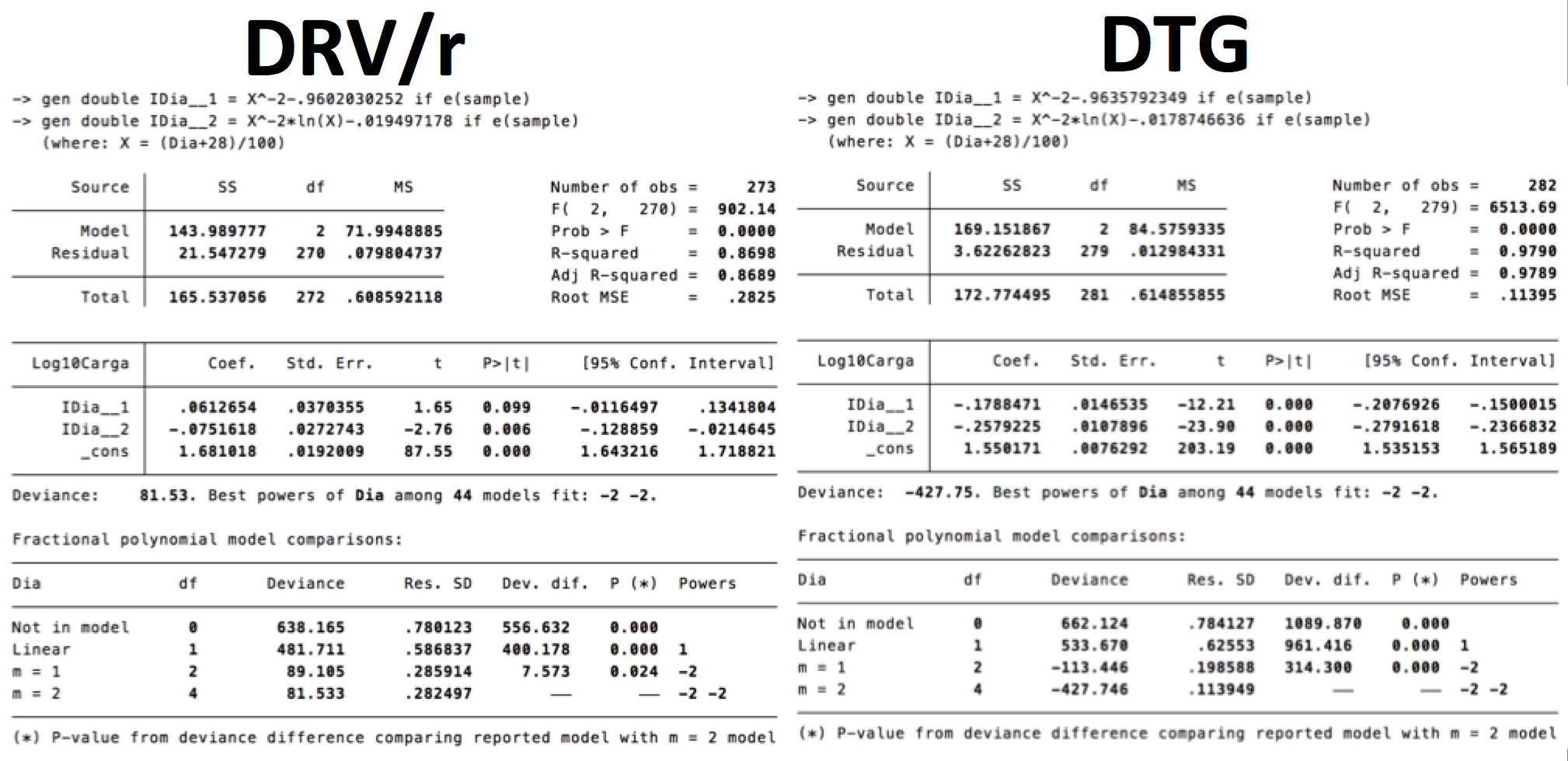


Figure U. DRVr treated patient. Simulated Log10 HIV-RNA over 24-weeks FLAMINGO <10,000 copies/mL (MEAN ± 95%CI)


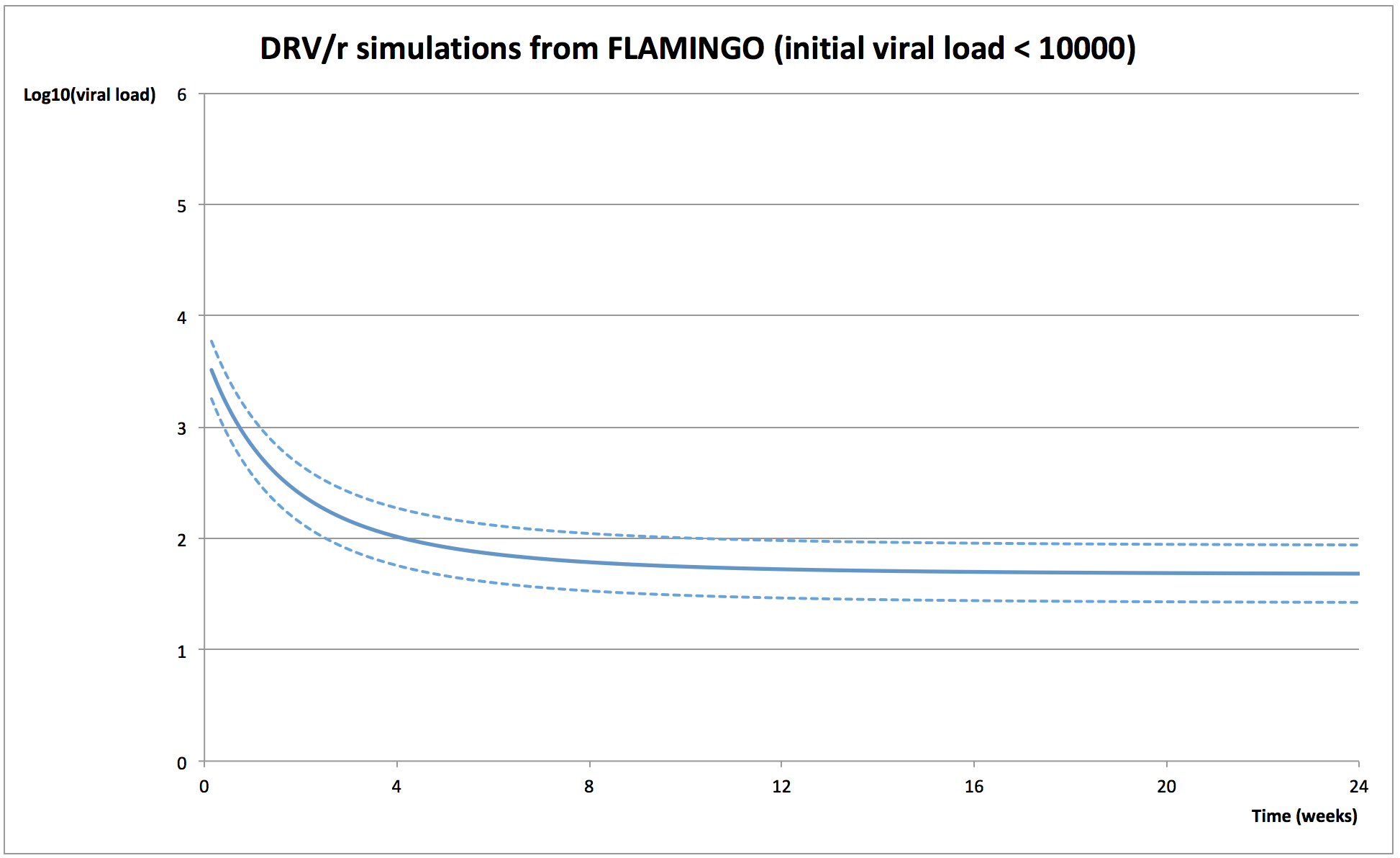


Figure V. DTG treated patient. Simulated Log10 HIV-RNA over 24-weeks FLAMINGO <10,000 copies/mL (MEAN ± 95%CI)


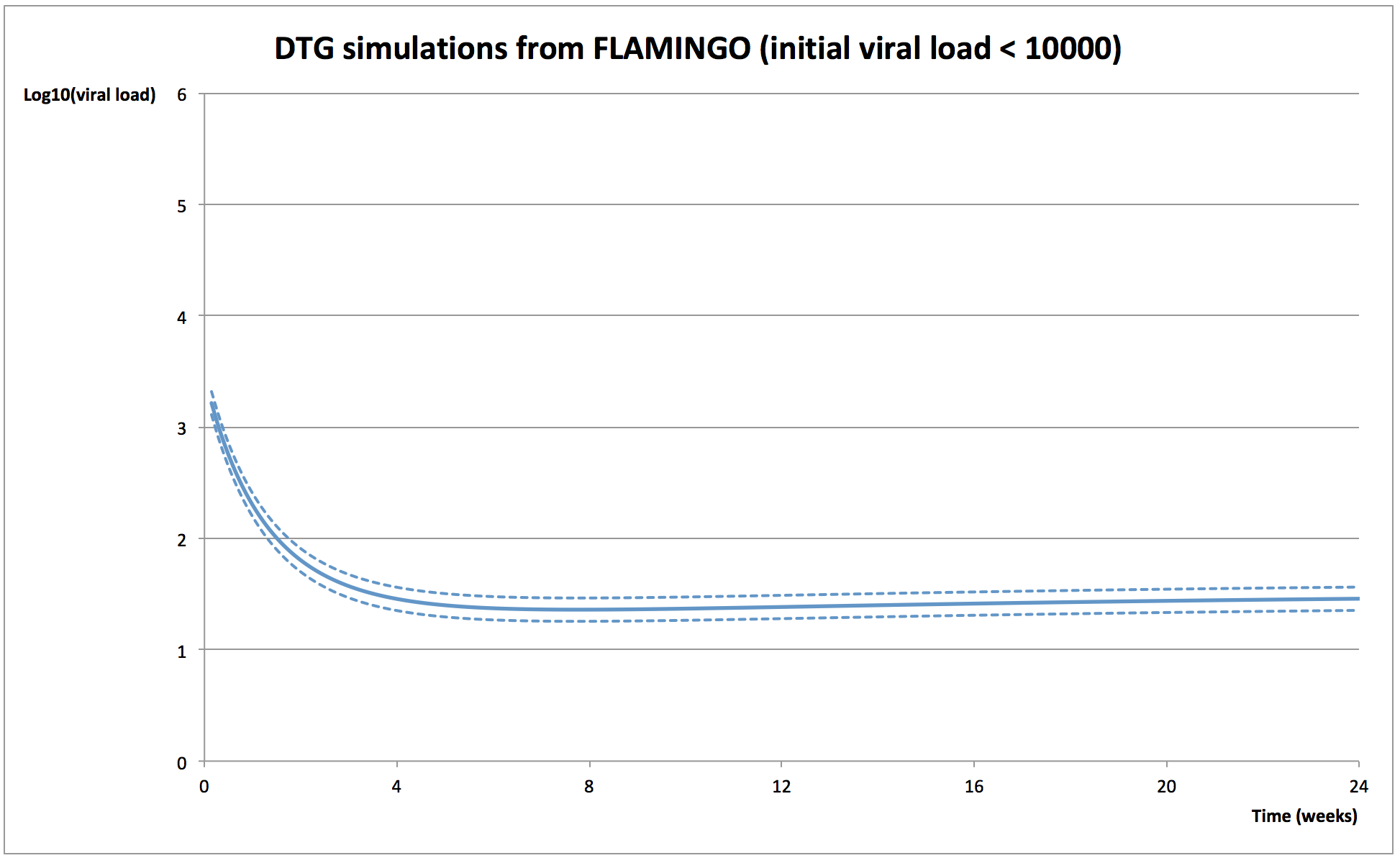


Figure W. STATA results for FLAMINGO ≥ 10,000 to <100,000 copies/mL


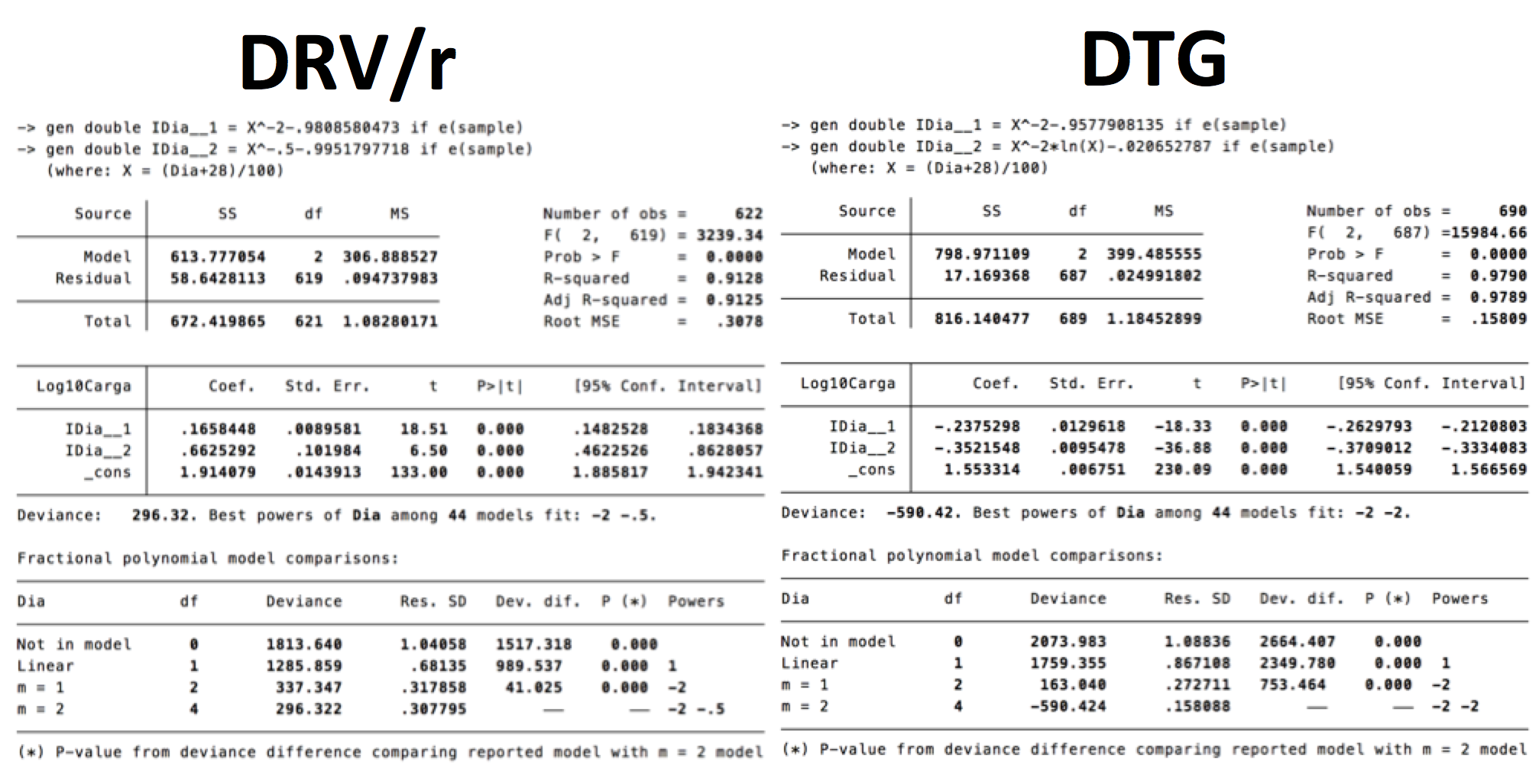


Figure X. DRVr treated patient. Simulated Log10 HIV-RNA over 24-weeks FLAMINGO ≥ 10,000 to <100,000 copies/mL (MEAN ± 95%CI)


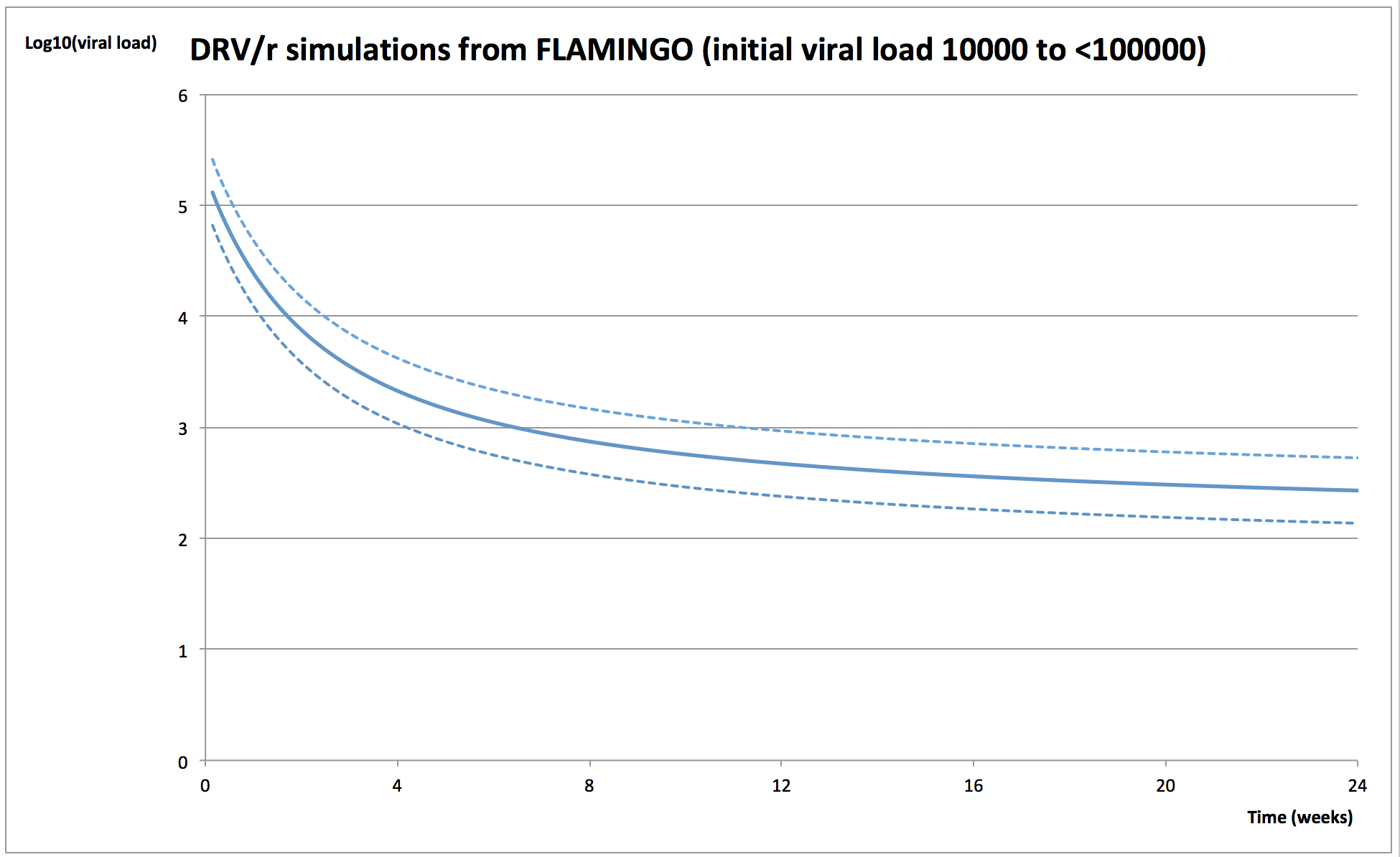


Figure Y. DTG treated patient. Simulated Log10 HIV-RNA over 24-weeks FLAMINGO ≥ 10,000 to <100,000 copies/mL (MEAN ± 95%CI)


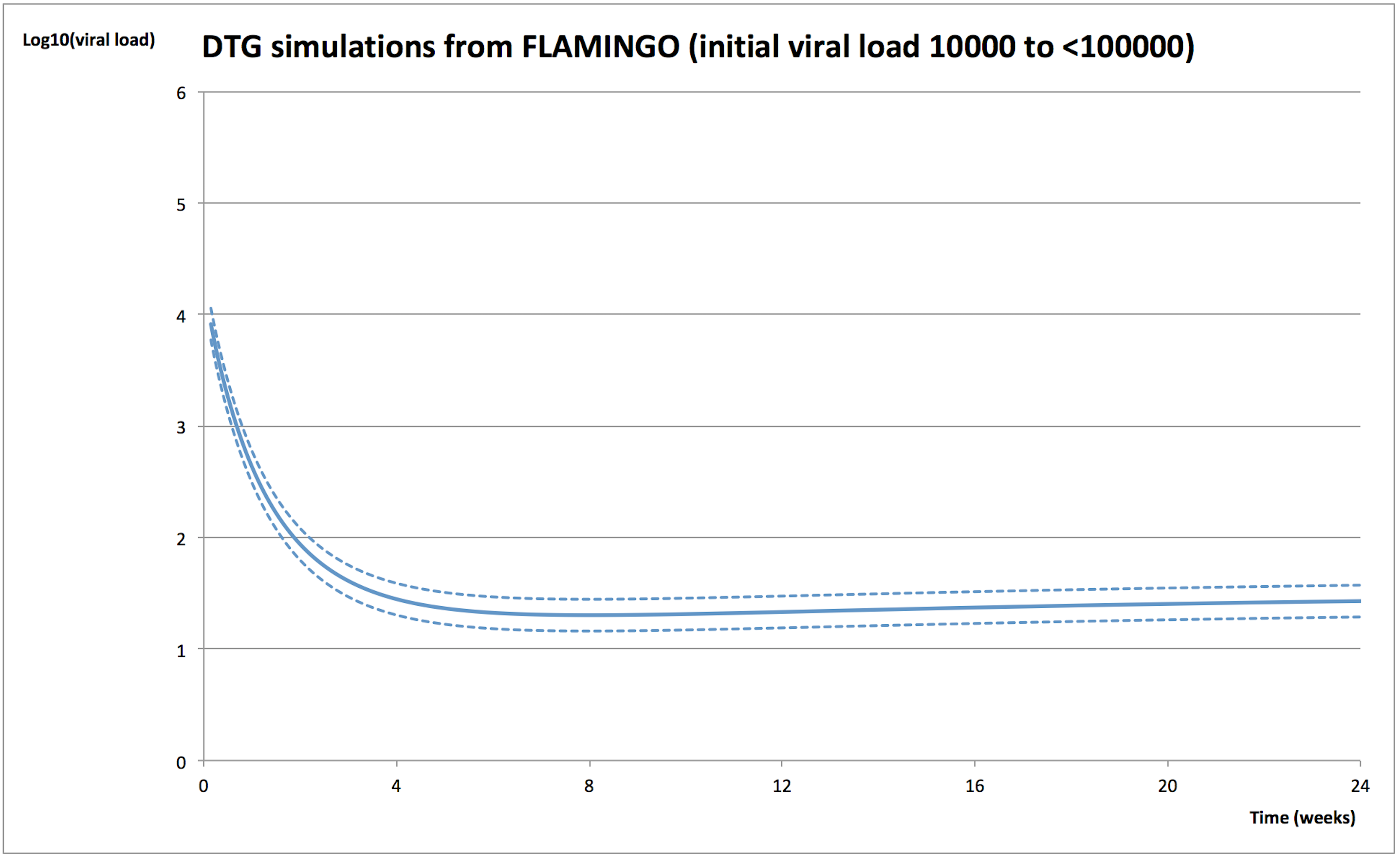


Figure Z. STATA results for FLAMINGO ≥ 100,000 copies/mL


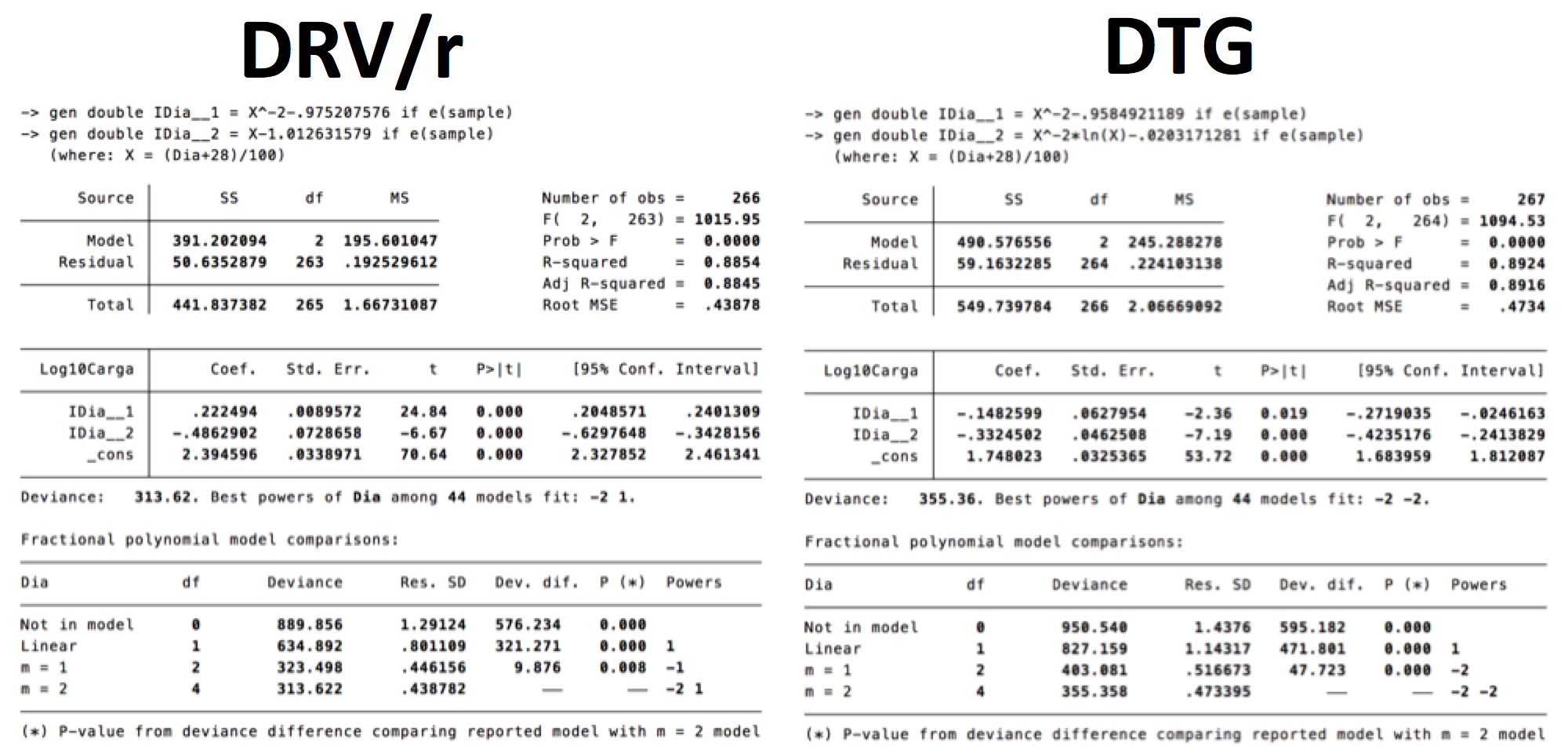


Figure AA. DRVr treated patient. Simulated Log10 HIV-RNA over 24-weeks DRVr FLAMINGO ≥ 100,000 copies/mL (MEAN ± 95%CI)


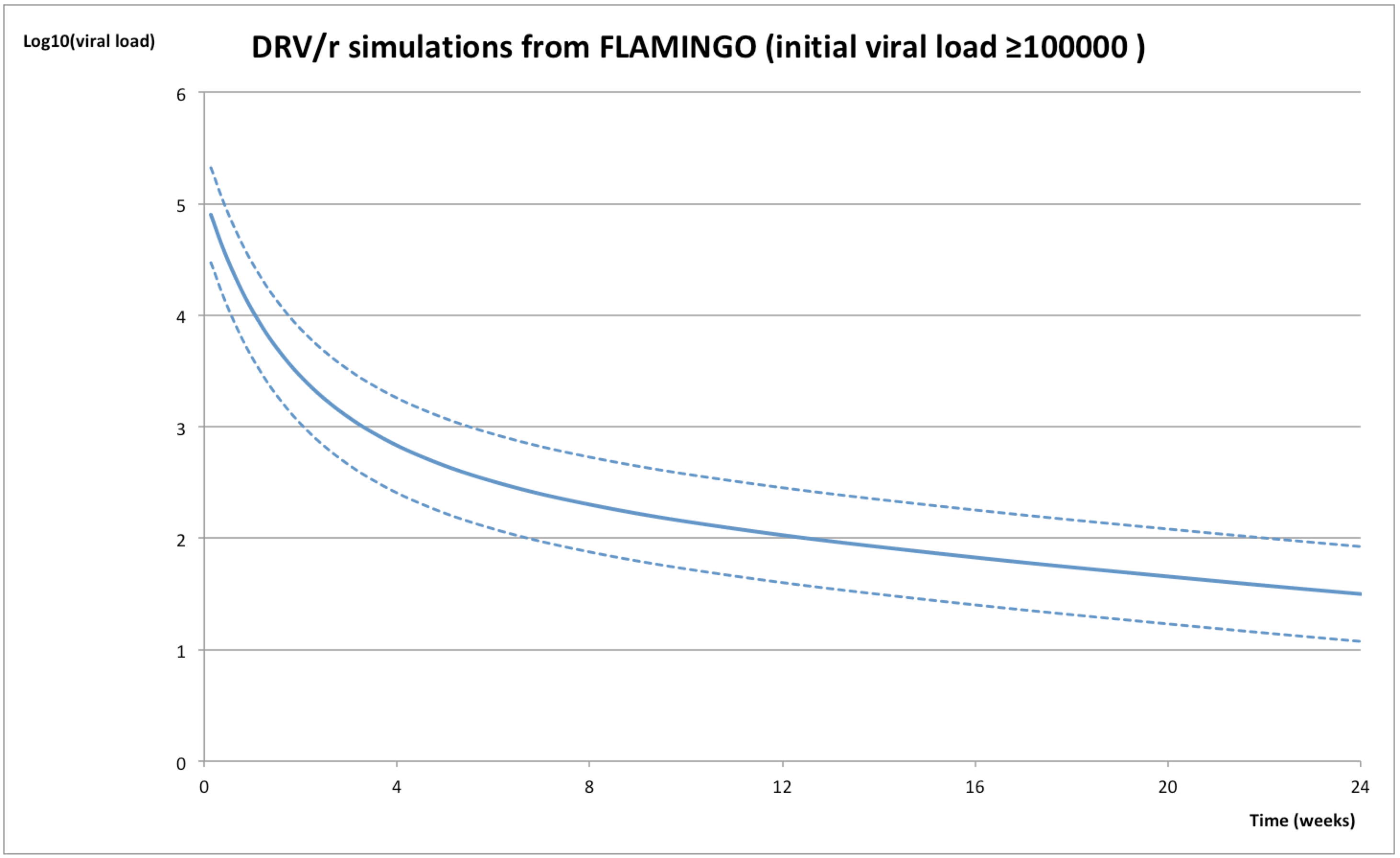


Figure AB. DTG treated patient. Simulated Log10 HIV-RNA over 24-weeks DTG FLAMINGO ≥ 100,000 copies/mL (MEAN ± 95%CI)


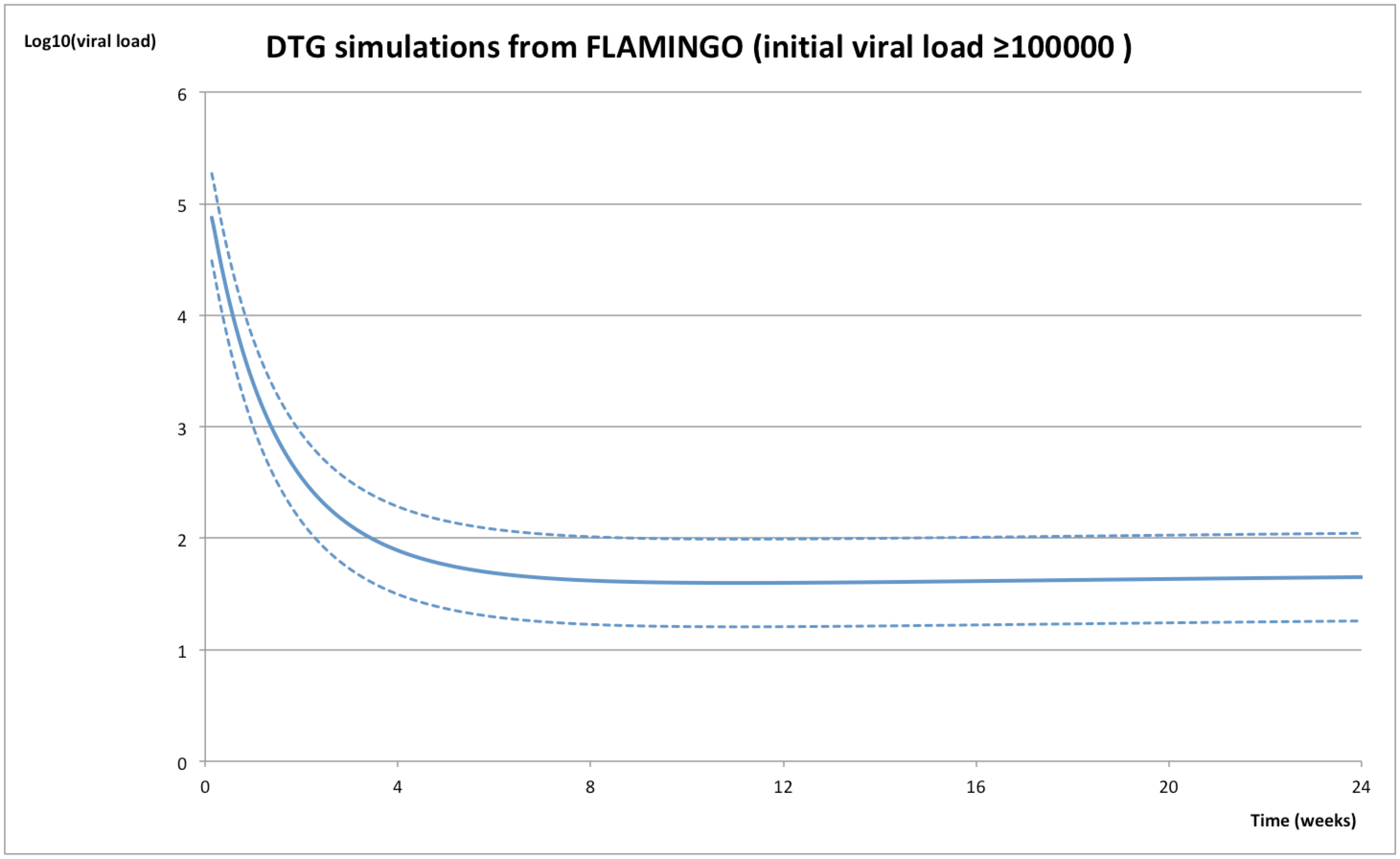


Figure AC. DTG treated patient. Simulated Log10 HIV-RNA over 24-weeks. Comparison of DTG MEAN in the three studies <10,000 copies/mL


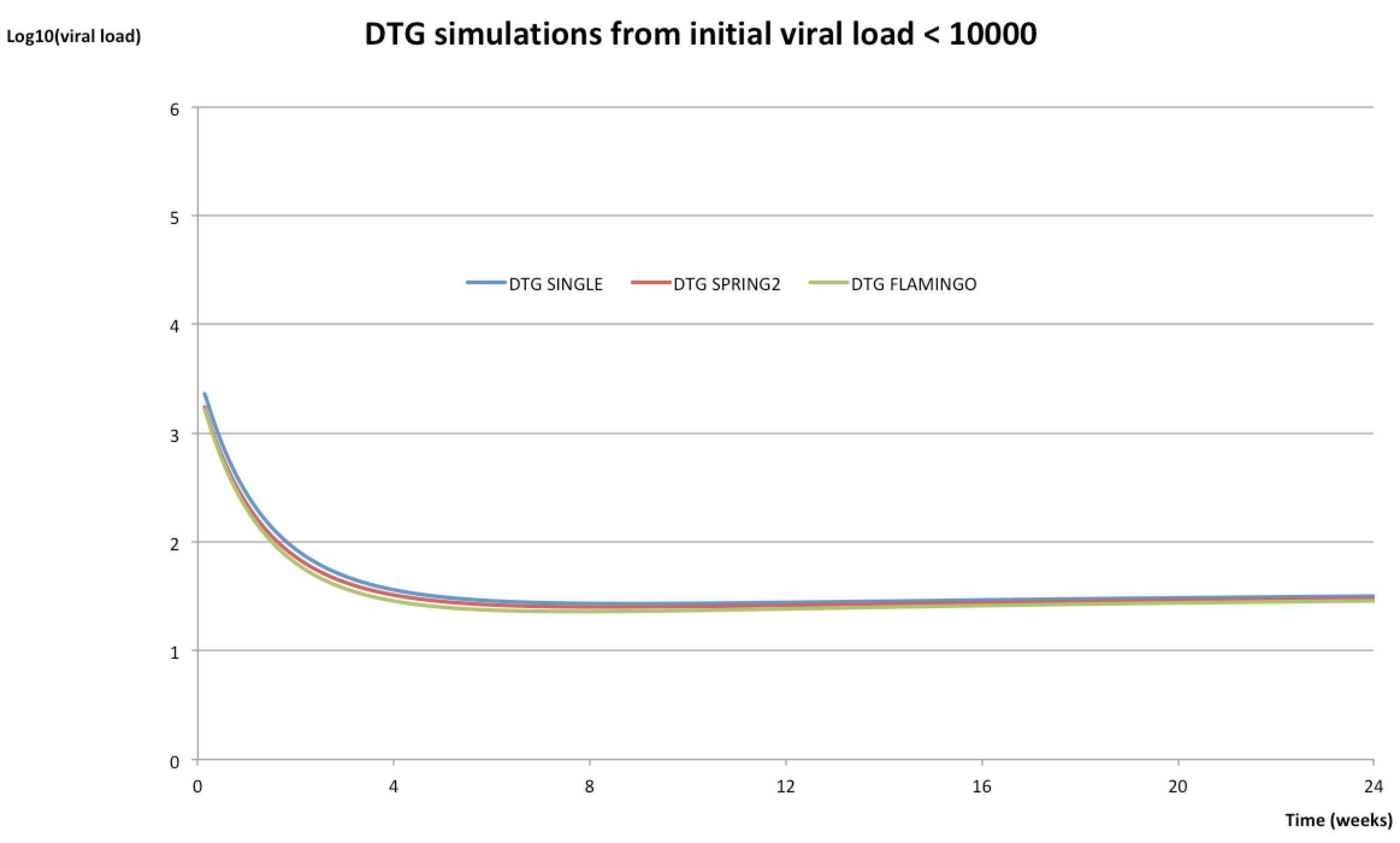


Figure AD. DTG treated patient. Simulated Log10 HIV-RNA over 24-weeks. Comparison of DTG MEAN in the three studies ≥ 10,000 to <100,000 copies/mL


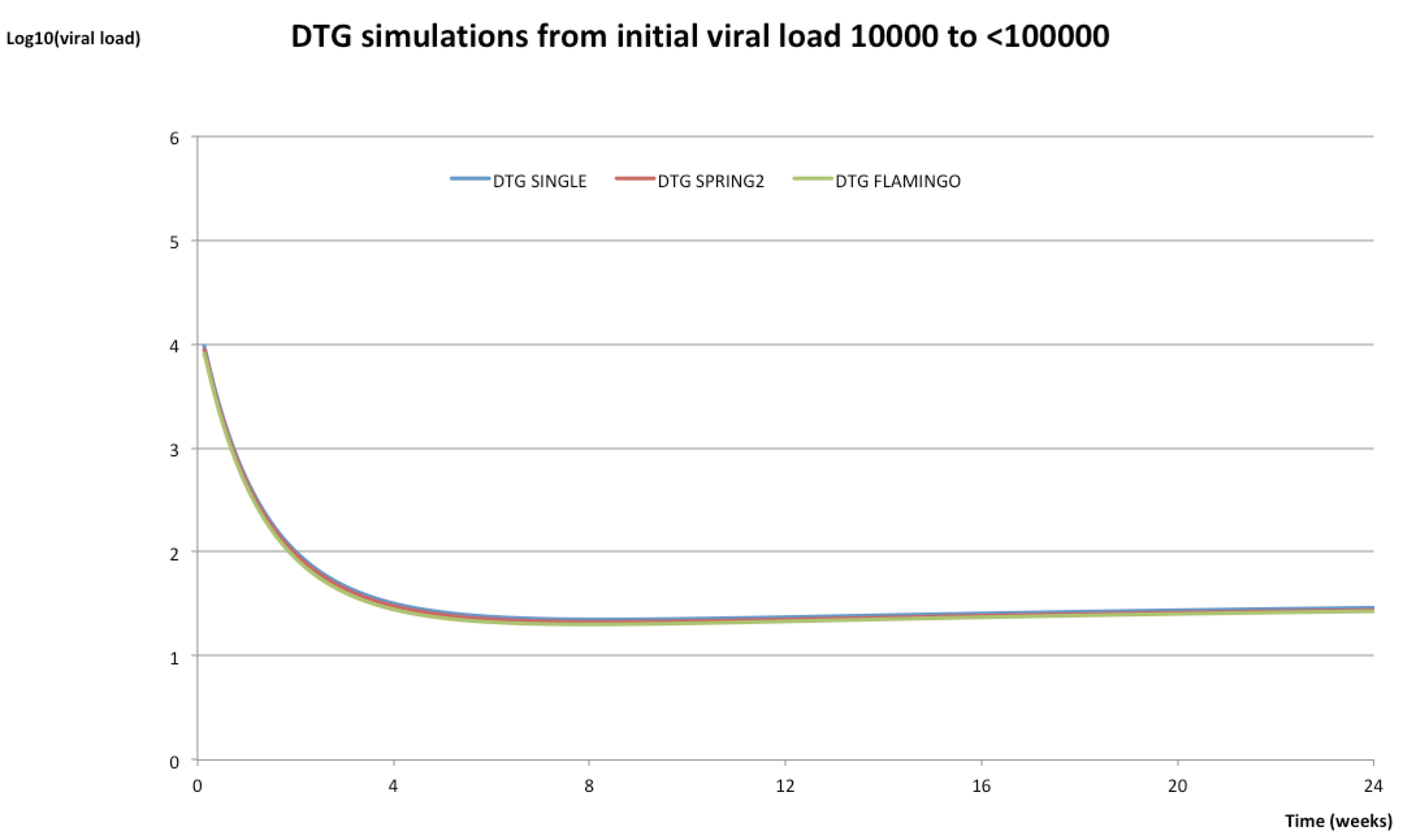


Figure AE. DTG treated patient. Simulated Log10 HIV-RNA over 24-weeks. Comparison of DTG MEAN in the three studies ≥ 100,000 copies/mL


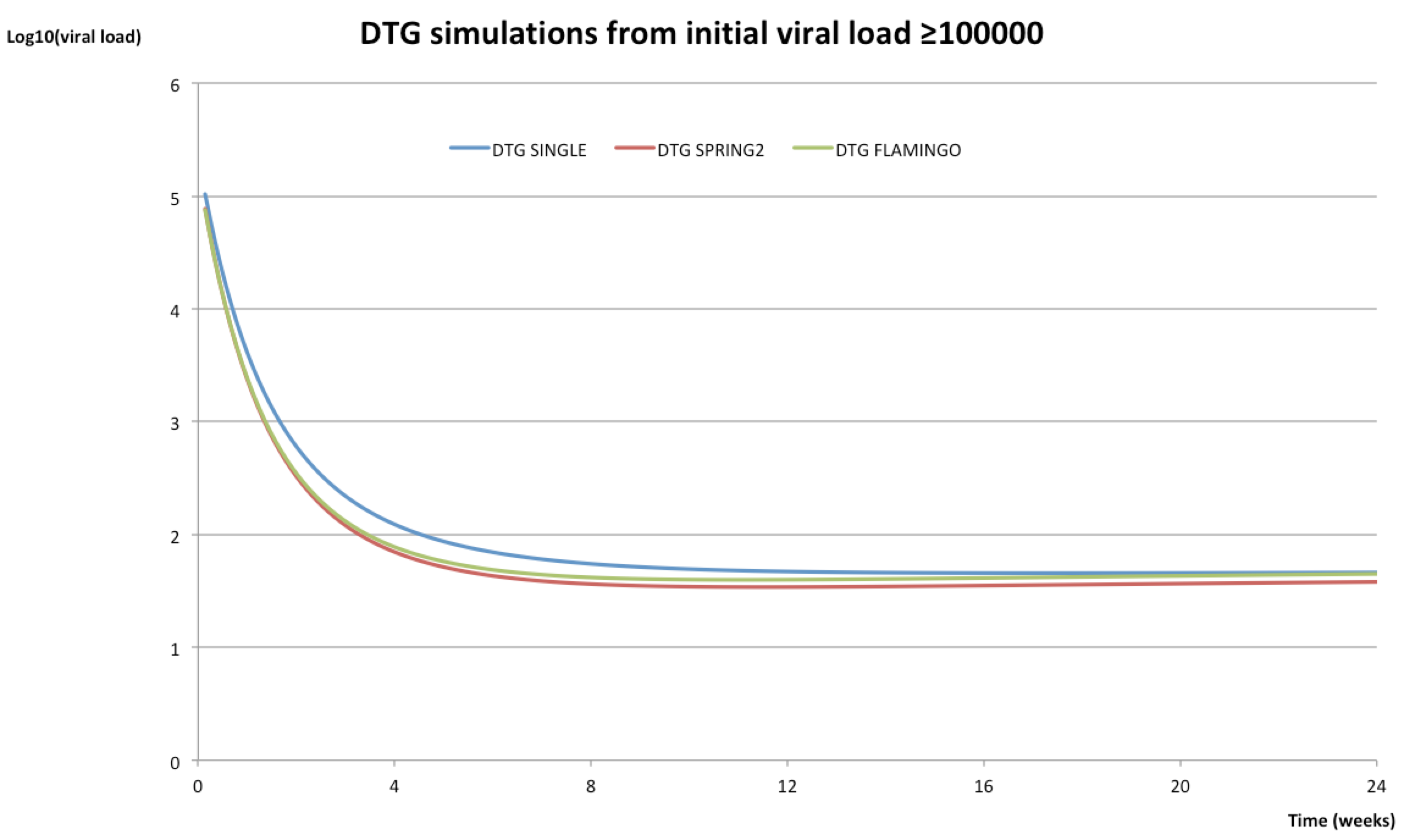


## Probability of infection

#### HIV RNA-dependent rates of infection by sexual act have been obtained from the Wilson mathematical model (9), in which the risk of transmission of HIV-1 according to HIV-RNA was modeled from the results of the Rakai study of HIV transmission in heterosexual couples [10]. On the basis of the Rakai data, each ten-fold increment in viral load is associated with a 2.45-fold (95% CI 1.85–3.26) increase in the risk of HIV transmission per sexual contact, as expressed by the equation:

β_1_=2.45^log^_10_^(V^_1_/^V^_0_^)^β_0_

#### where β_0_ is the probability of HIV transmission from a person with a baseline viral load V_0_, and β_1_ is the transmission probability corresponding to any other viral load V_1_, whether above or below the baseline.

#### V_0_ (lower and upper uncertainty bounds) is 4.3 x 10-5 (1.6 x 10-5 - 11.6 x 10-5) and corresponds to the expected transmission probability per male to female sexual act in a serodiscordant partnership, assuming the HIV-infected male has a viral load of 10 copies per ml.

#### As the Wilson equation used was done to estimate the risk of HIV transmission at a given HIV-1 RNA among serodiscordant heterosexual couples, the probability was modified by using the Odds Ratio of the type of sexual relationship (receptive or insertive anal intercourse) versus a receptive vaginal intercourse that were obtained from a recent systematic review by Patel et al. (Table 7) (10)

Table F. Estimated per-act probability of acquiring HIV-1 from an infected source, by sexual act

| **Activity** | **Risk per 10,000 exposures to an infected source** |
| --- | --- |
| Receptive Anal Sex | 138 |
| Insertive Anal Sex | 11 |
| Receptive Vaginal Sex | 8 |
| Insertive Vaginal Sex | 4 |

The odds of acquiring HIV-1 from an infected source taking into account receptive vaginal sex as a reference was 17.250 (138/8) times higher for receptive anal sex, and 1.375 (11/8) times higher for insertive anal sex.

Given the uncertainty of the β_0_ parameter in Wilson equation, a base case scenario has been developed using the Wilson β_0_ mean value. Additional sensitivity analyses were performed with the lower and upper uncertainty bounds.

## List of Scenarios Simulated (Base Case and Sensitivity Analyses)

Time horizon 8-weeks (Partners data from START study without any extrapolation)

### Base Case:

- - Probability of infection from β_0_ mean value.

### Sensitivity Analysis 1:

- - Probability of infection from β_0_ lower uncertainty bound value.

### Sensitivity Analysis 2:

- - Probability of infection from β_0_ upper uncertainty bound value.

## Model results

For each scenario and treatment evaluated, the model allows us to obtain:

- Number of simulated partners and average number of partners per patient.
- Number of simulated risk sexual encounters and number of simulated risk sexual encounters per partner.
- Number of simulated new infections (4 and 8 weeks)
- Number of patients needed to be treated with DTG instead of comparator (EFV/RAL/DRVr-based ART) to prevent one transmission event (NNT).

NNT is an expression of the number of patients a clinician would need to treat with a treatment option vs. another one, to prevent one additional adverse outcome or to obtain one additional benefit. This value depends on the condition, the intervention, the events, and the duration of follow-up ^(11)^. Therefore, it is believed that the NNT conveys both clinical and statistical significance to physicians and their patients in a single and easily comprehended measure.

NNT is the inverse of the absolute risk reduction (ARR) that is the absolute mathematical difference in outcome rates between treatment groups ^(11-13)^.

The interpretation is therefore that the lower the value of the NNT, the greater the number of patients who will benefit from the index treatment (DTG) compared to the comparator treatment.

Model outputs for the base case scenario and sensitivity analyses I to II are showed in the article, analyses III to VIII are summarized in **Tables 8 and 9**

**Table G.** Sensitivity analyses 3-5. Simulated sexual activity and HIV-1 transmission events after initiation of ART, for the full week 0 to 24 period, in the three treatment arms corresponding to the Single, Spring-2, and Flamingo trials parametrized according to the sexual risk behavior questionnaire in MSM recruited in the START trial^*#^

|  | **Sensitivity analysis 3** | | | **Sensitivity analysis 4** | | | **Sensitivity analysis 5** | | |
| --- | --- | --- | --- | --- | --- | --- | --- | --- | --- |
| **Simulated sexual activity** ^b^ | **Single** | **Spring-2** | **Flamingo** | **Single** | **Spring-2** | **Flamingo** | **Single** | **Spring-2** | **Flamingo** |
| Patients who initiated ART | 5,000,000 | 5,000,000 | 5,000,000 | 5,000,000 | 5,000,000 | 5,000,000 | 5,000,000 | 5,000,000 | 5,000,000 |
| Patients who engaged in CLS-D (20% of those initiating ART) | 1,000,000 | 1,000,000 | 1,000,000 | 1,000,000 | 1,000,000 | 1,000,000 | 1,000,000 | 1,000,000 | 1,000,000 |
| Partners of patients who engaged in CLS-D | 2,598,025 | 2,673,620 | 2,658,511 | 2,611,100 | 2,724,651 | 2,663.960 | 2,645,184 | 2,636,735 | 2,640,660 |
| Sexual encounters in patients who engaged in CLS-D | 21,915,417 | 21,299,208 | 21,603,651 | 21,892,955 | 21,323,595 | 21,640,506 | 22,022,190 | 21,607,188 | 21,738,984 |
| Partners per patient who engaged in CLS-D | 2.60 | 2.67 | 2.66 | 2.61 | 2.72 | 2.66 | 2.65 | 2.64 | 2.64 |
| Sexual encounters per partner in patients who engaged in CLS-D | 8.44 | 7.97 | 8.13 | 8.38 | 7.83 | 8.12 | 8.33 | 8.19 | 8.23 |
| **Simulated HIV-1 transmission events** | **No cART** | **No cART** | **No cART** | **No cART** | **No cART** | **No cART** | **No cART** | **No cART** | **No cART** |
| New infections | 1,463,825 | 1,405,224 | 1,351,224 | 1,186,779 | 1,170,695 | 197,236 | 1,618,000 | 1,532,087 | 1,532,087 |
| HIV-1 transmission events per 100 patients who initiated ART | 29.28 | 28.12 | 27.02 | 23.74 | 23.41 | 21.82 | 32.36 | 31.27 | 30.64 |
| HIV-1 transmission events per 100 patients who engaged in CLS-D | 56.34 | 52.56 | 50.84 | 45.45 | 42.97 | 7.40 | 61.17 | 58.02 | 58.02 |
| HIV-1 transmission events per 100 partners (CLS-D) | 1.46 | 1.41 | 1.35 | 1.19 | 1.17 | 0.20 | 1.62 | 1.53 | 1.53 |
|  | **DTG** | **DTG** | **DTG** | **DTG** | **DTG** | **DTG** | **DTG** | **DTG** | **DTG** |
| New infections | 25,995 | 21,029 | 22,965 | 9,944 | 7,495 | 8,414 | 69,999 | 59,543 | 61,793 |
| HIV-1 transmission events per 100 patients who initiated ART | 0.52 | 0.42 | 0.46 | 0.20 | 0.15 | 0.17 | 1.40 | 1.19 | 1.24 |
| HIV-1 transmission events per 100 patients who engaged in CLS-D | 1.00 | 0.79 | 0.86 | 0.38 | 0.28 | 0.32 | 2.65 | 2.26 | 2.34 |
| HIV-1 transmission events per 100 partners (CLS-D) | 0.03 | 0.02 | 0.02 | 0.01 | 0.01 | 0.01 | 0.07 | 0.06 | 0.06 |
| Proportion of HIV-1 transmission events compared with no Rx | 0.02 | 0.01 | 0.02 | 0.01 | 0.01 | 0.01 | 0.04 | 0.04 | 0.04 |
|  | **EFV** | **RAL** | **DRV/r** | **EFV** | **RAL** | **DRV/r** | **EFV** | **RAL** | **DRV/r** |
| New infections | 215,605 | 25,722 | 340,007 | 105,488 | 8.885 | 197,236 | 371,391 | 73,438 | 535,557 |
| HIV-1 transmission events per 100 patients who initiated ART | 4.31 | 0.51 | 6.80 | 2.11 | 0.18 | 3.94 | 7.43 | 1.47 | 10.71 |
| HIV-1 transmission events per 100 patients who engaged in CLS-D | 8.30 | 0.96 | 12.79 | 4.04 | 0.33 | 7.40 | 14.04 | 2.79 | 20.28 |
| HIV-1 transmission events per 100 partners (CLS-D) | 0.22 | 0.03 | 0.34 | 0.11 | 0.01 | 0.20 | 0.37 | 0.07 | 0.54 |
| Proportion of HIV-1 transmission events compared with no Rx | 0.15 | 0.02 | 0.25 | 0.09 | 0.01 | 0.18 | 0.23 | 0.05 | 0.35 |
| **NNT with DTG instead of comparator to prevent one infection** | **67** | **2,741** | **40** | **133** | **9,300** | **67** | **42** | **923** | **27** |

**Abbreviations**: cART, combination antiretroviral therapy; MSM, men who have sex with men; DTG, dolutegravir; EFV, efavirenz; DRV/r, darunavir/ritonavir; NNT, number needed to treat.

* These 24-weeks’ time horizon sensitivity analyses have been done assuming that, from the START data, those patients reporting only a single partner or two partners for the 8 weeks period, remain with the same number of partner during the 24 weeks period and for those reporting 3 or more partners, a third stayed with the same number of partners, another third multiplied by two the number of partners and the last third multiplied by three the number of partners.

^#^**Sensitivity analysis 3**: probability of transmission according to the mean value of the β_0_ parameter in the Wilson equation. **Sensitivity analysis 4**: probability of transmission according to the lower 95% confidence interval value of the β_0_ parameter in the Wilson equation. **Sensitivity analysis 5**: probability of transmission according to the upper 95% confidence interval value of the β_0_ parameter in the Wilson equation.

**^#^***A very small number of intercourse events among MSM in the START trial were reported to be with women*

**Table H.** Sensitivity analyses 6-8. Simulated sexual activity and HIV-1 transmission events after initiation of ART, for the full week 0 to 24 period, in the three treatment arms corresponding to the Single, Spring-2, and Flamingo trials parametrized according to the sexual risk behavior questionnaire in MSM recruited in the START trial^*#^

|  | **Sensitivity analysis 6** | | | **Sensitivity analysis 7** | | | **Sensitivity analysis 8** | | |
| --- | --- | --- | --- | --- | --- | --- | --- | --- | --- |
| **Simulated sexual activity** ^b^ | **Single** | **Spring-2** | **Flamingo** | **Single** | **Spring-2** | **Flamingo** | **Single** | **Spring-2** | **Flamingo** |
| Patients who initiated ART | 5,000,000 | 5,000,000 | 5,000,000 | 5,000,000 | 5,000,000 | 5,000,000 | 5,000,000 | 5,000,000 | 5,000,000 |
| Patients who engaged in CLS-D (20% of those initiating ART) | 1,000,000 | 1,000,000 | 1,000,000 | 1,000,000 | 1,000,000 | 1,000,000 | 1,000,000 | 1,000,000 | 1,000,000 |
| Partners of patients who engaged in CLS-D | 1,795,761 | 1,810,485 | 1,823,474 | 1,806,516 | 1,838,562 | 1,827,142 | 1,798,109 | 1,811,083 | 1,838,550 |
| Sexual encounters in patients who engaged in CLS-D | 22,892,166 | 21,997,701 | 22,045,167 | 22,974,378 | 22,723,509 | 22,213,269 | 22,684,062 | 22,036,893 | 22,182,246 |
| Partners per patient who engaged in CLS-D | 1.80 | 1.81 | 1.82 | 1.81 | 1.84 | 1.83 | 1.80 | 1.81 | 1.84 |
| Sexual encounters per partner in patients who engaged in CLS-D | 12.75 | 12.15 | 12.09 | 12.72 | 12.36 | 12.16 | 12.62 | 12.17 | 12.07 |
| **Simulated HIV-1 transmission events** | **No cART** | **No cART** | **No cART** | **No cART** | **No cART** | **No cART** | **No cART** | **No cART** | **No cART** |
| New infections | 1,465,824 | 1,410,904 | 1,366,836 | 1,248,795 | 1,182,508 | 1,064,757 | 1,588,500 | 1,548,513 | 1,538,711 |
| HIV-1 transmission events per 100 patients who initiated ART | 29.32 | 28.22 | 27.34 | 24.98 | 23.65 | 21.30 | 31.77 | 30.97 | 30.77 |
| HIV-1 transmission events per 100 patients who engaged in CLS-D | 81.63 | 77.93 | 74.96 | 69.13 | 64.32 | 58.27 | 88.34 | 85.50 | 83.69 |
| HIV-1 transmission events per 100 partners (CLS-D) | 1,47 | 1.41 | 1.37 | 1.25 | 1.18 | 1.06 | 1.59 | 1.55 | 1.54 |
|  | **DTG** | **DTG** | **DTG** | **DTG** | **DTG** | **DTG** | **DTG** | **DTG** | **DTG** |
| New infections | 30,346 | 24.021 | 25,372 | 11,001 | 9,161 | 9,530 | 77,497 | 64,568 | 67,334 |
| HIV-1 transmission events per 100 patients who initiated ART | 0.61 | 0.48 | 0.51 | 0.22 | 0.18 | 0.19 | 1.55 | 1.29 | 1.35 |
| HIV-1 transmission events per 100 patients who engaged in CLS-D | 1.69 | 1.33 | 1.39 | 0.61 | 0.50 | 0.52 | 4.31 | 3.57 | 3.66 |
| HIV-1 transmission events per 100 partners (CLS-D) | 0.03 | 0.02 | 0.03 | 0.01 | 0.01 | 0.01 | 0.08 | 0.06 | 0.07 |
| Proportion of HIV-1 transmission events compared with no Rx | 0.02 | 0.02 | 0.02 | 0.01 | 0.01 | 0.01 | 0.05 | 0.04 | 0.04 |
|  | **EFV** | **RAL** | **DRV/r** | **EFV** | **RAL** | **DRV/r** | **EFV** | **RAL** | **DRV/r** |
| New infections | 231,037 | 29,768 | 350,396 | 113,533 | 11,494 | 206,993 | 386,714 | 81,877 | 543,383 |
| HIV-1 transmission events per 100 patients who initiated ART | 4.62 | 0.60 | 7.01 | 2.27 | 0.23 | 4.14 | 7.73 | 1.64 | 10.87 |
| HIV-1 transmission events per 100 patients who engaged in CLS-D | 12.87 | 1.64 | 19.22 | 6.28 | 0.63 | 11.33 | 21.51 | 4.52 | 29.55 |
| HIV-1 transmission events per 100 partners (CLS-D) | 0.23 | 0.03 | 0.35 | 0.11 | 0.01 | 0.21 | 0.39 | 0.08 | 0.54 |
| Proportion of HIV-1 transmission events compared with no Rx | 0.16 | 0.02 | 0.26 | 0.09 | 0.01 | 0.19 | 0.24 | 0.05 | 0.35 |
| **NNT with DTG instead of comparator to prevent one infection** | **44** | **1,533** | **27** | **87** | **3,790** | **45** | **29** | **509** | **19** |

**Abbreviations**: cART, combination antiretroviral therapy; MSM, men who have sex with men; DTG, dolutegravir; EFV, efavirenz; DRV/r, darunavir/ritonavir; NNT, number needed to treat.

*These 24-weeks’ time horizon sensitivity analyses have been done assuming the same partners as the START study and assuming that the 3-fold increase in duration is extrapolated to multiply by three the number of risky sexual encounters.

^#^**Sensitivity analysis 6**: probability of transmission according to the mean value of the β_0_ parameter in the Wilson equation. **Sensitivity analysis 7**: probability of transmission according to the lower 95% confidence interval value of the β_0_ parameter in the Wilson equation. **Sensitivity analysis 8**: probability of transmission according to the upper 95% confidence interval value of the β_0_ parameter in the Wilson equation.

**^#^***A very small number of intercourse events among MSM in the START trial were reported to be with women*

# References:

1. Walmsley SL, Antela A, Clumeck N, Duiculescu D, Eberhard A, Gutierrez F, et al. Dolutegravir plus abacavir-lamivudine for the treatment of HIV-1 infection. The New England journal of medicine. 2013;369(19):1807-18.

2. Raffi F, Rachlis A, Stellbrink HJ, Hardy WD, Torti C, Orkin C, et al. Once-daily dolutegravir versus raltegravir in antiretroviral-naive adults with HIV-1 infection: 48 week results from the randomised, double-blind, non-inferiority SPRING-2 study. Lancet (London, England). 2013;381(9868):735-43.

3. Clotet B, Feinberg J, van Lunzen J, Khuong-Josses MA, Antinori A, Dumitru I, et al. Once-daily dolutegravir versus darunavir plus ritonavir in antiretroviral-naive adults with HIV-1 infection (FLAMINGO): 48 week results from the randomised open-label phase 3b study. Lancet (London, England). 2014;383(9936):2222-31.

4. Rodger AJ, Lampe FC, Grulich AE, Fisher M, Friedland G, Phanuphak N, et al. Transmission risk behaviour at enrolment in participants in the INSIGHT Strategic Timing of AntiRetroviral Treatment (START) trial. HIV medicine. 2015;16 Suppl 1:64-76.

5. Committee UKCHCW. Predicting virological decay in patients starting combination antiretroviral therapy. AIDS (London, England). 2016;30(11):1817-27.

6. Chen R, Huang Y. Mixed-Effects Models with Skewed Distributions for Time-Varying Decay Rate in HIV Dynamics. Communications in statistics: Simulation and computation. 2016;45(2):737-57.

7. Doung H, Volding D. Modelling continuous risk variables: Introduction to fractional polynomial regression. VJS. 2014;1(2):5.

8. Royston P, Altman DG. Regression Using Fractional Polynomials of Continuous Covariates: Parsimonious Parametric Modelling. Journal of the Royal Statistical Society Series C (Applied Statistics). 1994;43(3):429-67.

9. Wilson DP, Law MG, Grulich AE, Cooper DA, Kaldor JM. Relation between HIV viral load and infectiousness: a model-based analysis. Lancet (London, England). 2008;372(9635):314-20.

10. Patel P, Borkowf CB, Brooks JT, Lasry A, Lansky A, Mermin J. Estimating per-act HIV transmission risk: a systematic review. AIDS (London, England). 2014;28(10):1509-19.

11. Cordell WH. Number needed to treat (NNT). Annals of emergency medicine. 1999;33(4):433-6.

12. Flechner L, Tseng TY. Understanding results: P-values, confidence intervals, and number need to treat. Indian journal of urology : IJU : journal of the Urological Society of India. 2011;27(4):532-5.

13. Mendes D, Alves C, Batel-Marques F. Number needed to treat (NNT) in clinical literature: an appraisal. BMC medicine. 2017;15(1):112.
